# Evaluating Climate Change Effects on Swan Habitats Within China: Adaptive Strategies for Sustainable Conservation

**DOI:** 10.1002/ece3.72238

**Published:** 2025-10-01

**Authors:** Ke Zhang, Jun Lin, Jianghua Zheng, Xuan Li, Li Xu, Liang Liu, Xuan Liu, Xi Jin, Rong Fu, Xinwei Wang, Yunzhi Sang, Xiaoyu Guo

**Affiliations:** ^1^ College of Geography and Remote Sensing Science Xinjiang University Urumqi China; ^2^ Xinjiang Key Laboratory of Oasis Ecology Urumqi China; ^3^ Center for Wildlife Conservation and Monitoring of Xinjiang Uygur Autonomous Region Urumqi China

**Keywords:** climate change, habitat suitability, maximum entropy model, protected area, swans

## Abstract

Under the combined pressures of global climate change and human activities, swan habitats in China are facing severe threats, posing direct challenges to the effectiveness of existing protected areas. However, the dynamic changes in the distribution of swan habitats and conservation coverage under climate change remain insufficiently explored. In this study, we focused on three widely distributed swan species in China— *Cygnus cygnus* (Whooper Swan), *Cygnus columbianus* (Bewick's Swan), and *Cygnus olor* (Mute Swan). Using the maximum entropy (MaxEnt) model, we projected the spatial distribution patterns of their habitats and the trends in conservation rates under recent period (2001–2020) and future periods (2021–2040 and 2041–2060) across three Shared Socioeconomic Pathways (SSP1–2.6, SSP2–4.5, and SSP5–8.5). The results indicated that the MaxEnt model performed well, with mean values of training and testing area under the receiver operating characteristic curve (AUC) of 0.966 and 0.956, respectively, and a mean true skill statistic (TSS) of 0.833 across all periods. Slope, NDVI, proximity to water, and isothermality (Bio3) were identified as the key environmental variables jointly influencing the distribution of the three swan species. During the recent period, the total suitable habitat areas of the three swan species were 44.89 × 10^4^, 54.18 × 10^4^, and 48.33 × 10^4^ km^2^, respectively. Under future scenarios, overall habitat ranges remained relatively stable, but the suitability structure shifted, with low suitability habitats showing greater fluctuations. In terms of conservation coverage, the overall conservation rate of suitable habitats for all three species remained below 11%. Coverage of highly suitable habitats was higher than that of moderately and low suitable habitats, but protection gaps persisted, especially for Whooper Swan and Bewick's Swan. These findings highlight significant mismatches between swan habitats and reserve networks and provide a scientific basis for optimizing conservation planning and adaptive management under climate change.

## Graphical abstract

We modeled the distributions of Whooper, Bewick's, and Mute swans in China using the MaxEnt model under recent (2001–2020) and future climate scenarios (2021–2040, 2041–2060), identifying slope, NDVI, proximity to water, and isothermality as key drivers. Results show that while overall habitat ranges remain relatively stable, low‐suitability areas expand and conservation coverage stays below 11%, revealing significant protection gaps that call for optimized reserve networks under climate change.

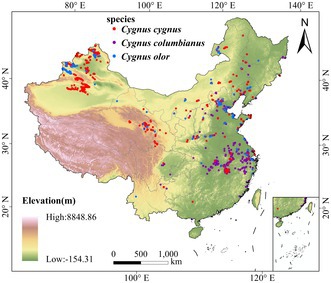

## 1 Introduction

As a major source of ecological pressures on human well‐being in the Anthropocene, climate change is driving habitat loss, fragmentation, and degradation worldwide (Abrahms 2021; Abrahms et al. 2023; Banks‐Leite et al. 2020). Habitat loss is widely recognized as the leading driver of species extinction (Luedtke et al. 2023; Sandor et al. 2022; Staude et al. 2020), and climate change has emerged as a key factor accelerating this process (Arneth et al. 2020; Chase et al. 2020). As global climate patterns change rapidly, species are forced to alter their distributions in search of suitable habitats (Li and Park 2020). Migratory birds are especially vulnerable because their life cycles rely on at least two geographically distinct habitats (Gross 2024; Runge et al. 2014). To adapt, migratory birds may adjust their migration patterns to keep key life cycle events synchronized with suitable habitat conditions in space and time (Linssen et al. 2023). Such adaptations are crucial for their survival and reproduction. Therefore, assessing the impacts of climate change on migratory bird habitats is essential for identifying potential range shifts. This knowledge also provides the foundation for evidence‐based conservation strategies and adaptive management.

As the largest country in Asia, China encompasses vast territories, diverse climates, and rich ecosystems, playing a vital role in global climate change mitigation and biodiversity conservation (Mi et al. 2023). Since establishing its first nature reserve in 1956, China has steadily strengthened environmental laws and implemented large‐scale ecological restoration projects (Li and Pimm 2020). As a Party to the Convention on Biological Diversity (CBD), China hosted the 15th meeting of the Conference of the Parties to the United Nations CBD (COP15) in 2021. It has also actively promoted international cooperation in biodiversity conservation (Li and Pimm 2020; Ma 2023). However, under climate change, migratory bird habitats worldwide remain at severe risk of degradation and loss (Culp et al. 2017; La Sorte et al. 2017; Lisovski et al. 2024). To address this challenge, China released the Action Plan for the Conservation and Restoration of Migratory Bird Flyways (2024–2030) in June 2024. The plan emphasizes strengthening the protection and restoration of key habitats and integrating the nature reserve system. Given the diverse ecological requirements and habitat preferences of migratory bird species, species‐specific conservation strategies have become essential for enhancing conservation effectiveness. Such tailored approaches contribute to achieving long‐term biodiversity goals in a rapidly changing environment.

China lies along four of the world's nine major migratory bird flyways, providing critical stopover habitats for numerous species (Ma et al. 2019). Among these migratory birds, swans ( *Cygnus cygnus* , *Cygnus columbianus* , and *Cygnus olor* ) are widely distributed, long‐distance migratory waterfowl (Rees et al. 2019). All three belong to the genus *Cygnus* (family *Anatidae*, order *Anseriformes*) and are classified as Class II protected species under China's List of National Key Protected Wild Animals. Swan populations are highly sensitive to environmental change, with their distributions and numbers shaped by climate, pollutants, and human disturbance (Wood et al. 2019). In recent years, swans have increasingly been observed in the same regions (Ao et al. 2020; Meng et al. 2020). This pattern may reflect improved ecological conditions, but it may also signal shifts driven by complex pressures such as climate change (Culp et al. 2017; Liu et al. 2018). Swans require high‐quality habitats and are widely recognized as both indicator and flagship species of wetland ecosystems (Bauer and Hoye 2014; Chen et al. 2023; Johnsgard 2020; Ogden et al. 2014). Given their unique ecological and conservation value, swans are also regarded as umbrella species that provide broad conservation benefits (Holopainen et al. 2022).

Protected areas (PAs) are the cornerstone of efforts to curb global biodiversity loss. They provide critical refugia for species affected by climate change, supporting adaptation and resilience (Li et al. 2024; Watson et al. 2014; Xu et al. 2022). Although the number and extent of PAs have expanded rapidly worldwide, biodiversity decline has not been effectively mitigated (Visconti et al. 2019). A global assessment of 1451 migratory bird species found that only 9% received comprehensive protection along their migratory routes (Runge et al. 2015). The same study revealed that the loss of stopover sites is a key driver of population declines along flyways, and China is no exception (Runge et al. 2015). In response, China has established multiple protected areas that buffer habitat from climate change (Wang et al. 2025). Although both the number and area of PAs have increased—achieving some success in protecting waterbirds and their habitats (Wauchope et al. 2022)—the overall conservation rate has not improved accordingly (Li and Pimm 2020). For migratory species with dynamic distributions, static PA boundaries are insufficient to accommodate climate‐driven habitat shifts (Wilson et al. 2025). Other studies suggest that the proportion of key habitats covered by reserves in China will remain low in the long term (Liang et al. 2018). Therefore, urgent adjustments and updates to China's reserve network are needed. Future reserve expansion should focus not only on increasing area but also on optimizing geographical placement (Li and Pimm 2020). Although some studies have evaluated the conservation rates of migratory birds in China under climate change at a macro scale (Liang et al. 2021; Zhang, Wei, and Xu 2023), species‐specific analyses of habitat distribution and reserve coverage remain limited. This gap makes it difficult to account for the unique ecological characteristics and conservation needs of key species.

The MaxEnt model is a machine learning framework based on information theory. It estimates a probability distribution of species presence that maximizes entropy subject to environmental constraints (Capera‐Aragones et al. 2023). This principle enables MaxEnt to capture relationships between species occurrence and environmental variables, making it a widely used tool for species distribution modeling. The model is particularly suitable for dealing with presence—only or uneven species distribution data (Jiménez‐Valverde et al. 2008; Phillips and Dudík 2008), which is critical for assessing wildlife habitat suitability. The risk of overfitting can be reduced by tuning the regularization multiplier during model training (Hartl et al. 2024; Tibshirani 2011). Numerous studies have demonstrated that MaxEnt model achieves high accuracy and reliability in predicting bird distributions (Liang et al. 2021; Wang et al. 2024; Wauchope et al. 2017; Zhu et al. 2022).

Therefore, this study focuses on three swan species— *Cygnus cygnus* , *Cygnus columbianus* , and *Cygnus olor* —to systematically assess habitat dynamics and conservation coverage under climate change. Using the MaxEnt model with bioclimatic and other environmental variables, the study projected and analyzed suitable habitat distributions and their protection status. Specifically, the study aims to: (1) identify the key environmental variables shaping swan habitat distributions; (2) assess spatiotemporal trends of suitable habitats under recent and future climate scenarios; and (3) quantify the coverage of existing protected areas and highlight potential conservation gaps. The results provide a scientific basis for understanding how swan species respond to environmental change and offer guidance for optimizing the spatial configuration of nature reserves and strengthening long‐term habitat conservation and adaptive management.

## 2 Materials and Methods

### 2.1 Acquisition and Preprocessing of Swan Distribution Records

We obtained the swan occurrence data used in this study from the three primary sources. (1) Tracking data from Xinjiang, collected legally in 2021–2022 by the Center for Wildlife Conservation and Monitoring of Xinjiang Uygur Autonomous Region and the National Bird Banding Center of China, and authorized for scientific use by our research team. Tracking was conducted using a GPS‐GSM satellite system, which included information on the swans' geographic location, time, flight altitude, speed, and communication signal strength. The dataset included satellite tracking records of 110 individual swans from the Xinjiang region, comprising 92 *Cygnus cygnus* and 18 *Cygnus olor* . To ensure that the data used in the MaxEnt model objectively reflected the natural activity states of swans, tracking records from the first 3 days after device deployment were excluded (Linssen et al. 2023). In addition, only stationary records (speed = 0) with high signal quality (grades A or B) were retained, thereby excluding in‐flight locations to focus on habitat use rather than movement trajectories. (2) Public observation records were obtained from the Global Biodiversity Information Facility (GBIF, https://www.gbif.org/) and the China Birdwatching Record Center (https://www.birdreport.cn/). The temporal coverage of the records was 2004–2024 for Whooper Swans, 2006–2023 for Bewick's Swans, and 2007–2024 for Mute Swans. (3) Field surveys were conducted by our team from 1 to 10 January 2024 in Yining County, Bole City, Tacheng Prefecture, and Manas County (Xinjiang). These surveys primarily recorded occurrences of Whooper Swans and Mute Swans. To minimize disturbance to the swans, the team maintained a safe distance, used high‐powered binoculars for long‐range observations, and recorded swan locations with GPS devices combined with satellite imagery (Sauer 2002).

**FIGURE 1:**
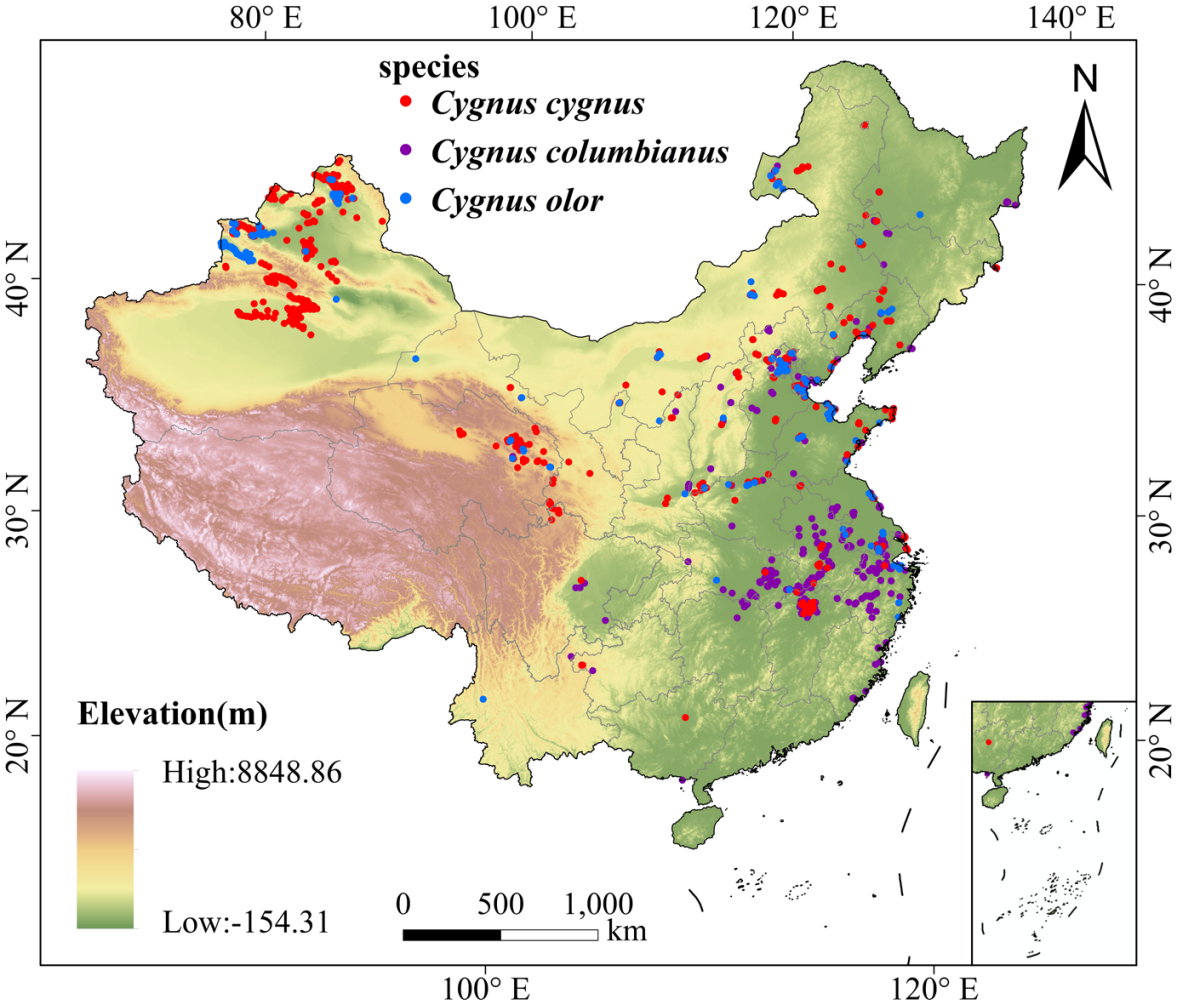
Overview of the study area and occurrence records of three swan species: *Cygnus cygnus* , *Cygnus columbianus* , and *Cygnus olor* . Red, purple, and blue points represent the three species, respectively.

Through the three approaches described above, a total of 424,563 distribution records of the three swan species in China were obtained including 421,570 tracking records, 2978 public records, and 15 field survey records. To better capture the ecological requirements and distribution characteristics of each species, the three swans were processed and analyzed separately. Because of the diverse data sources and inconsistent formats—and especially the potential spatial bias and misidentification in public databases—data quality issues could affect the accuracy of species distribution models (SDMs) (Koo et al. 2025). Therefore, we applied rigorous standardization and quality control including harmonizing coordinate systems and time formats, checking metadata, and removing duplicate or anomalous records. We also verified spatial locations using Google Earth and excluded records located in clearly erroneous sites such as urban buildings (Yang et al. 2025). In addition, to correct for sampling bias and reduce spatial autocorrelation, a spatial thinning approach was applied, retaining only one record within each 1 × 1 km grid cell (Brown et al. 2017; Liu, Xu, et al. 2024). After this procedure, the final number of occurrence records was 632 for Whooper Swans, 581 for Bewick's Swans, and 234 for Mute Swans (Figure 1, Table S1). Detailed information on the distribution records of the three swan species is provided in Table S2.

### 2.2 Selection of Environmental Variables

To ensure that the environmental variables were ecologically relevant to swan distributions, this study selected five categories of predictors: bioclimate, topography, vegetation, human activity, and water sources. Together, these variables represent the principal drivers shaping the distribution patterns of swans (Gayet et al. 2011; Wilson et al. 2025; Yan et al. 2024). Bioclimatic data were obtained from WorldClim 2.1 (https://worldclim.org/) (Fick and Hijmans 2017). We downloaded historical monthly climate data for 2001–2020 (2.5 arc‐minutes) including average minimum temperature (°C), average maximum temperature (°C), and total precipitation (mm). Nineteen standard bioclimatic variables for the recent period were derived using the “biovars” function in the R package dismo (Hijmans et al. 2023). Future bioclimatic data were derived from three general circulation models (GCMs): BCC‐CSM2‐MR, MIROC6, and HadGEM3‐GC31‐LL (Imani wa Rusaati and Won Kang 2024; Tatebe et al. 2019) (Table S3). The data covered two time periods, the 2030s (2021–2040) and the 2050s (2041–2060). Species distributions were modeled separately with each GCM, and results were averaged as an ensemble mean to integrate predictions, thereby improving reliability and reducing single‐model bias in habitat suitability estimates (Luo et al. 2024). We considered three Shared Socioeconomic Pathways (SSPs): SSP1–2.6 (low‐carbon emissions, optimistic development), SSP2–4.5 (moderate‐carbon emissions, balanced development), and SSP5–8.5 (high‐carbon emissions, unsustainable development) (O'Neill et al. 2017). Combining the three SSPs with two time periods yielded six future climate scenarios.

Elevation data (DEM) for China was obtained from the Shuttle Radar Topography Mission (SRTM) dataset with a spatial resolution of 1 km (https://earthexplorer.usgs.gov/). Slope and aspect layers were derived from the DEM using ArcGIS.

The Normalized Difference Vegetation Index (NDVI) is a key metric for assessing vegetation health and productivity, and it strongly influences food availability for swans (Echeverría‐Caro et al. 2022). The NDVI data used in this study were derived from the MOD13A3 monthly dataset (https://www.usgs.gov/) with a spatial resolution of 1 km (Didan 2015). The time span was set to 2001–2020, during which annual data were extracted using the maximum value composite method. The 20‐year mean was then calculated and used as the vegetation factor variable.

Human Footprint data were obtained from the Global Human Footprint dataset (1‐km resolution, 2001–2020; https://figshare.com/) (Mu et al. 2022). This dataset integrates eight key variables, including social factors, agricultural production, transportation, and nighttime lights, thereby providing a comprehensive representation of global human activity and human‐induced pressures. The average Human Footprint index for 2001–2020 was calculated and used as the human activity environmental variable.

In this study, Dis‐water was defined as the Euclidean distance (m) from each analysis grid cell to the nearest surface water. Smaller values indicate closer proximity to water. Here, “surface water” refers to pixels classified as permanent wetlands or water bodies in the land cover/land use data products. Dis‐water was constructed for the recent (2001–2020) and future periods as follows. For the recent period, this study used the MCD12Q1 land cover dataset from the International Geosphere‐Biosphere Program (IGBP) with a spatial resolution of 500 m (https://www.usgs.gov/) (Friedl et al. 2019). From this dataset, pixels classified as permanent wetlands and water bodies that remained unchanged during 2001–2020 were extracted, merged, and applied to calculate Euclidean distances, generating the Dis‐water environmental variable for the recent period. For the future periods, the analysis employed the global 1 km land use simulation dataset for 2020–2060, which follows the IGBP classification scheme and reflects dynamic land‐use changes under different SSP climate scenarios (Hou et al. 2022). For the 2030s (2021–2040) and 2050s (2041–2060), pixels classified as permanent wetlands and water bodies were extracted from the simulation dataset. Euclidean distance rasters were then generated using the same method as for the recent period, producing the future Dis‐water variables.

The boundary data of nature reserves were obtained from the World Database on Protected Areas (WDPA, https://protectedplanet.net; updated 20 June 2025). Considering the incomplete coverage of protected areas in China within the WDPA, this study supplemented the dataset with national nature reserve data released by the Resource and Environmental Science Data Platform of the Chinese Academy of Sciences (http://www.resdc.cn) to ensure spatial completeness and accuracy (Figure S1). In addition, the official directory of nature reserves published by the Ministry of Ecology and Environment of the People's Republic of China (www.mee.gov.cn) was used for verification.

To ensure consistency in modeling, we standardized the boundaries and coordinate systems of all raster data. We then resampled their spatial resolution to 1 km using bilinear interpolation. Detailed descriptions of all environmental variables, including their classification, definitions, and measurement units, are provided in Table S4.

### 2.3 Model Establishment and Evaluation

#### 2.3.1 Model Establishment

To reduce the impact of autocorrelation and multicollinearity among environmental variables on model predictions, Spearman's rank correlation coefficients were first calculated in SPSS (Figure S2). When the correlation coefficient between two variables was |*r*| ≥ 0.75, the variable with a higher contribution, as determined by Jackknife analysis, was retained while the redundant factor was removed (Liu, Xu, et al. 2024; Song et al. 2025; Wu et al. 2025). After screening, 14 environmental variables were retained for Whooper Swan, 10 for Tundra Swan, and 12 for Mute Swan (Table S5).

Before running the models, the ENMeval package was used to optimize parameters for each species and reduce the risk of overfitting from default MaxEnt settings. These parameters included the Feature Combination (FC) and the Regularization Multiplier (RM), which were optimized to improve model performance and predictive accuracy (Liu, Xu, et al. 2024; Xu et al. 2024). For FCs, we considered the five basic features provided by the MaxEnt model: Linear (L), Quadratic (Q), Hinge (H), Product (P), and Threshold (T). These features were configured into six different FCs: H, L, LQ, LQH, LQHP, and LQHPT. The range of the RM was set between 0.5 and 4 with an interval of 0.5, resulting in 48 distinct parameter settings (Zhao et al. 2021). Finally, the optimal MaxEnt parameters were determined for each species: Whooper Swan, FC = LQHPT and RM = 0.5; Bewick's Swan, FC = LQHPT and RM = 1; Mute Swan, FC = LQHPT and RM = 1.5. During model execution, 75% of the occurrence records were used as the training set and 25% as the test set. The number of background points was set to 10,000, and each MaxEnt run was replicated 10 times per climate scenario.

#### 2.3.2 Model Evaluation

We evaluated the model performance using the Receiver Operating Characteristic (ROC) curve. The predictive accuracy was assessed based on the area under the ROC curve (AUC) and the True Skill Statistic (TSS). AUC values range from 0 to 1, and TSS values from −1 to 1. Higher values of both AUC and TSS indicate greater predictive accuracy of the model (Allouche et al. 2006; Xu et al. 2024).

### 2.4 Classification of Suitable Habitat Levels

Based on the prediction results of the MaxEnt model, the “maximum training sensitivity plus specificity” logistic threshold (MTSS) was adopted as the cutoff. Using the SDM tools in ArcGIS, the continuous suitability outputs were quantified and classified into four levels: unsuitable habitats (< MTSS), low suitable habitats (MTSS—0.4), moderately suitable habitats (0.4–0.6), and highly suitable habitats (0.6–1). The MTSS threshold maximizes the sum of sensitivity and specificity of the training data while minimizing species omission errors, thereby ensuring both the ecological conservatism and comparability of habitat classifications. It is one of the most widely applied and robust thresholding methods in species distribution modeling (Liu et al. 2013; Wang et al. 2019; Xu et al. 2024).

### 2.5 Evaluation of Suitable Habitat Conservation Levels

The coverage of suitable habitats by protected areas is widely regarded as one of the key indicators for assessing the effectiveness of protected area networks (Liang et al. 2021; Liu, Li, et al. 2024; Shi et al. 2023; Yang et al. 2025). To evaluate the conservation levels for the three swan species within China's existing protected areas, we performed a spatial overlay analysis between the habitat distribution maps predicted by the MaxEnt model for different periods and the boundary data of China's nature reserves. Specifically, we calculated the proportion of each suitability class (high, moderate, low) located within protected areas relative to its total area, which was defined as the conservation rate by suitability class. We then calculated the proportion of the total area of all suitable habitats covered by protected areas relative to the overall suitable habitat area, which was defined as the total conservation rate of each species.

In addition, to assess the robustness of the total conservation rate under different thresholds, two commonly used alternative thresholds were introduced in addition to the MTSS threshold applied in the main analysis. The first was the 10th percentile training presence threshold (10% TP), which allows a certain proportion of occurrence points to be treated as noise or potential location errors, and is therefore relatively liberal. The second was the equal sensitivity and specificity threshold (ESS), which balances sensitivity and specificity and is relatively conservative. These two thresholds have been widely recommended and applied in species distribution modeling studies (Liu et al. 2016, 2013; Merow et al. 2013).

## 3 Results

### 3.1 Assessing Model Performance

We constructed the optimized MaxEnt models separately for the three swan species. Model performance was reported as mean values with ranges in parentheses. For Whooper Swan, training AUC was 0.964 (0.962–0.968), test AUC was 0.950 (0.947–0.957), and TSS was 0.838 (0.831–0.858). For Bewick's Swan, training AUC was 0.963 (0.962–0.965), test AUC was 0.957 (0.956–0.961), and TSS was 0.831 (0.827–0.846). For Mute Swan, training AUC was 0.971 (0.969–0.978), test AUC was 0.961 (0.956–0.968), and TSS was 0.830 (0.813–0.869) (Table 1). Across different climate scenarios and time periods, all models achieved AUC > 0.94 and TSS > 0.8, which indicated high discriminatory power and stable performance.

**TABLE 1:** MaxEnt model evaluation results (AUC and TSS) for three swan species under different Shared Socioeconomic Pathways and time periods.

Species	Shared Socioeconomic Pathways	Period	AUC (train)	AUC (test)	TSS
Whooper Swan	—	Recent	0.968	0.952	0.858
	SSP1–2.6	2030s	0.962	0.948	0.831
		2050s	0.963	0.952	0.833
	SSP2–4.5	2030s	0.963	0.947	0.834
		2050s	0.963	0.957	0.834
	SSP5–8.5	2030s	0.964	0.947	0.841
		2050s	0.963	0.949	0.835
Bewick's Swan	—	Recent	0.965	0.961	0.846
	SSP1–2.6	2030s	0.962	0.956	0.828
		2050s	0.962	0.957	0.831
	SSP2–4.5	2030s	0.962	0.956	0.827
		2050s	0.962	0.957	0.829
	SSP5–8.5	2030s	0.963	0.958	0.830
		2050s	0.962	0.957	0.827
Mute Swan	—	Recent	0.978	0.968	0.869
	SSP1–2.6	2030s	0.971	0.963	0.830
		2050s	0.969	0.963	0.821
	SSP2–4.5	2030s	0.971	0.960	0.830
		2050s	0.969	0.959	0.823
	SSP5–8.5	2030s	0.971	0.961	0.826
		2050s	0.969	0.956	0.813

### 3.2 Identification of Key Environmental Variables

**FIGURE 2:**
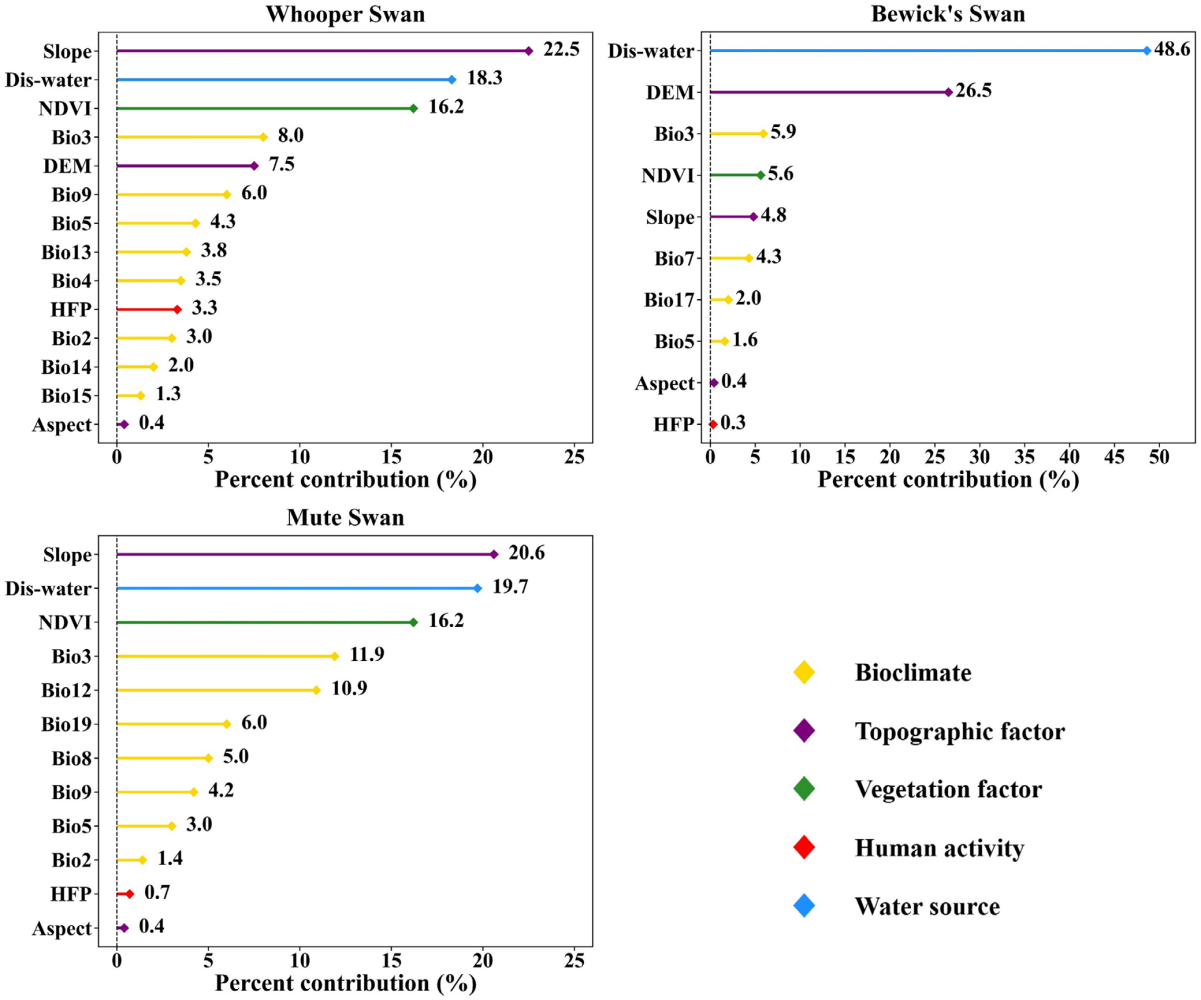
Contribution rates of environmental variables in the MaxEnt models of the three swan species during the recent period. Different colors indicate variable categories.

Based on MaxEnt model outputs for the recent period, we evaluated the relative importance of environmental variables in predicting suitable habitats of the three swan species. The contribution rate of variables was used to measure the relative contribution of each variable during model training, which reflected its strength in explaining species distributions. When the cumulative contribution rate of several variables exceeds 85%, they can be considered the key environmental variables influencing the distribution of the species (Yang et al. 2022). For Whooper Swan model, the key variables were slope (22.5%), Dis‐water (18.3%), NDVI (16.2%), isothermality (Bio3, 8.0%), elevation (7.5%), mean temperature of the driest quarter (Bio9, 6.0%), maximum temperature of the warmest month (Bio5, 4.3%), and precipitation of the wettest month (Bio13, 3.8%). Temperature seasonality (Bio4), Human Footprint, and mean diurnal range (Bio2) also made minor contributions. The Bewick's Swan model showed concentrated dependence on a few variables, with Dis‐water (48.6%), elevation (26.5%), Bio3 (5.9%), and NDVI (5.6%) together exceeding 85%. Other variables contributed less than 5%. For Mute Swan, the key environmental variables included slope (20.6%), Dis‐water (19.7%), NDVI (16.2%), Bio3 (11.9%), annual precipitation (Bio12, 10.9%), and precipitation of the coldest quarter (Bio19, 6.0%) (Figure 2).

### 3.3 Distribution of Suitable Habitats for the Three Swan Species During the Recent Period

**FIGURE 3:**
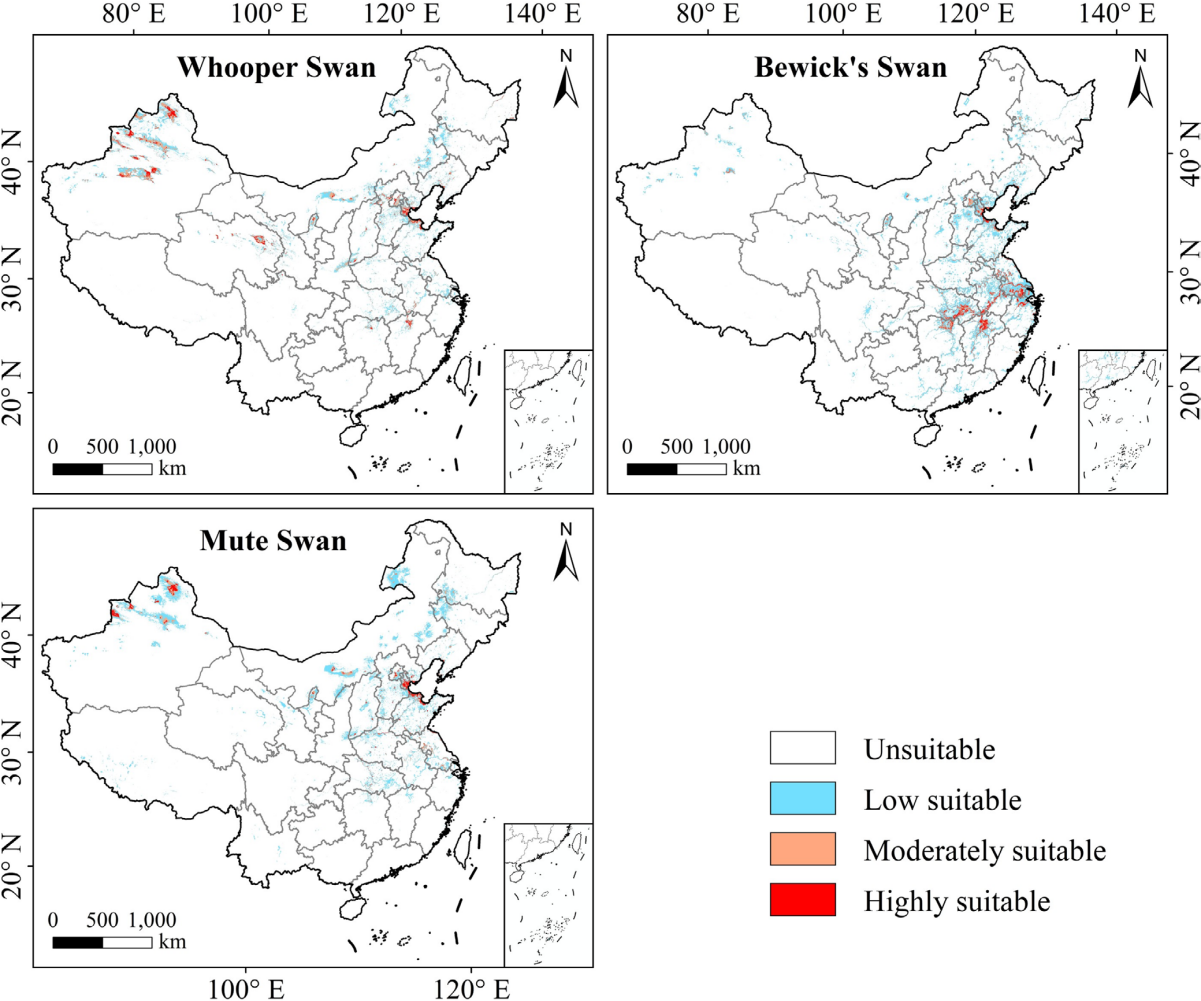
Spatial distribution of suitable habitats for the three swan species in China during the recent period.

Under the recent climate conditions, the three swan species exhibited distinct patterns of suitable habitat distribution (Figure 3). Suitable habitats for Whooper Swan and the Mute Swan were mainly concentrated in northern Xinjiang, the North China Plain, and the middle and lower reaches of the Yellow River, with core distribution areas in Beijing, Hebei, and Shandong. In addition, central Xinjiang also represented an important habitat for Whooper Swan. The total suitable habitat areas of the two species were comparable, at approximately 44.89 × 10^4^ km^2^ for Whooper Swan and 48.33 × 10^4^ km^2^ for Mute Swan. Highly suitable habitats accounted for 7.96% (3.58 × 10^4^ km^2^) and 7.02% (3.39 × 10^4^ km^2^), respectively, while moderately suitable habitats covered 27.21% (12.22 × 10^4^ km^2^) and 15.43% (7.46 × 10^4^ km^2^). In contrast, Bewick's Swan had a broader distribution range. Beyond the North China Plain, its suitable habitats extended across the middle and lower Yangtze Plain, particularly in Hubei, Hunan, Jiangxi, Anhui, and Jiangsu. Its total suitable habitat area reached 54.18 × 10^4^ km^2^, with highly suitable habitats accounted for 8.84% (4.79 × 10^4^ km^2^)—the highest among the three species. However, low suitable habitats dominated, which represented more than 70% of its total (38.88 × 10^4^ km^2^).

### 3.4 Trends in Suitable Habitat Changes Under Future Climate Scenarios

**FIGURE 4:**
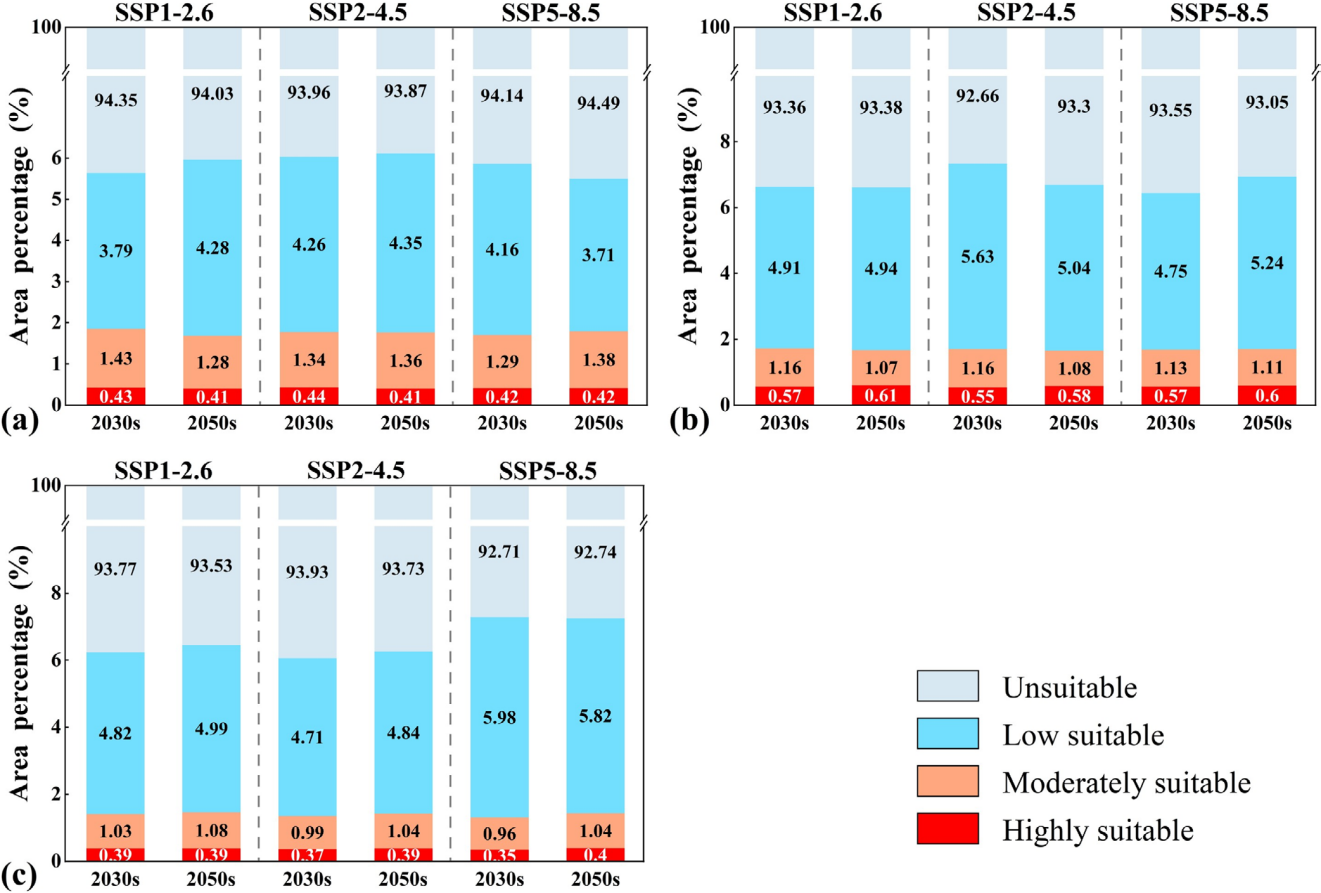
Proportions of suitable habitat areas at different suitability levels for the three swan species in China during the 2030s and 2050s under future climate scenarios. Panels (a–c) represent the Whooper Swan, Bewick's Swan, and Mute Swan, respectively.

Under different future climate scenarios, the overall spatial distribution patterns of suitable habitats for the three swan species remained broadly similar to those in the recent period, without showing notable regional shifts (Figure S3). However, in terms of suitability classes, low suitability habitats exhibited more pronounced fluctuations and expansion trends (Figure 4). For Whooper Swan, the proportions of highly suitable and moderately suitable habitats remained relatively stable (highly suitable: 0.41%–0.44%; moderately suitable: 1.28%–1.43%). By contrast, low suitability habitats increased under SSP1–2.6 and SSP2–4.5, and reached 4.28% (41.07 × 10^4^ km^2^) and 4.35% (41.80 × 10^4^ km^2^) in the 2050s, respectively, while showing only a slight decrease under SSP5–8.5 (Figure 4a). For Bewick's Swan, the proportion of highly suitable habitats showed a slight increase, whereas moderately suitable habitats exhibited a minor declining trend. Low suitability habitats fluctuated more strongly; under SSP2–4.5, they decreased from 5.63% (54.02 × 10^4^ km^2^) in the 2030s to 5.04% (48.83 × 10^4^ km^2^) in the 2050s, with a net change of about 0.6% (5.59 × 10^4^ km^2^) (Figure 4b). For Mute Swan, the proportions of highly and moderately suitable habitats consistently remained between 1.31% and 1.47%. In contrast, low suitability habitats expanded under SSP1–2.6 and SSP2–4.5, whereas under SSP5–8.5 they exhibited a slight contraction, declined from 5.98% (57.43 × 10^4^ km^2^) in the 2030s to 5.82% (55.84 × 10^4^ km^2^) in the 2050s (Figure 4c).

In addition, this study further quantified the prediction uncertainty of habitat area for the three swan species under different suitability classes in future periods. The mean and standard deviation of the predicted areas were reported, and the coefficient of variation (CV) was calculated. Detailed results are provided in Table S6.

### 3.5 Conservation Rates of Suitable Habitats for the Three Swan Species

**FIGURE 5:**
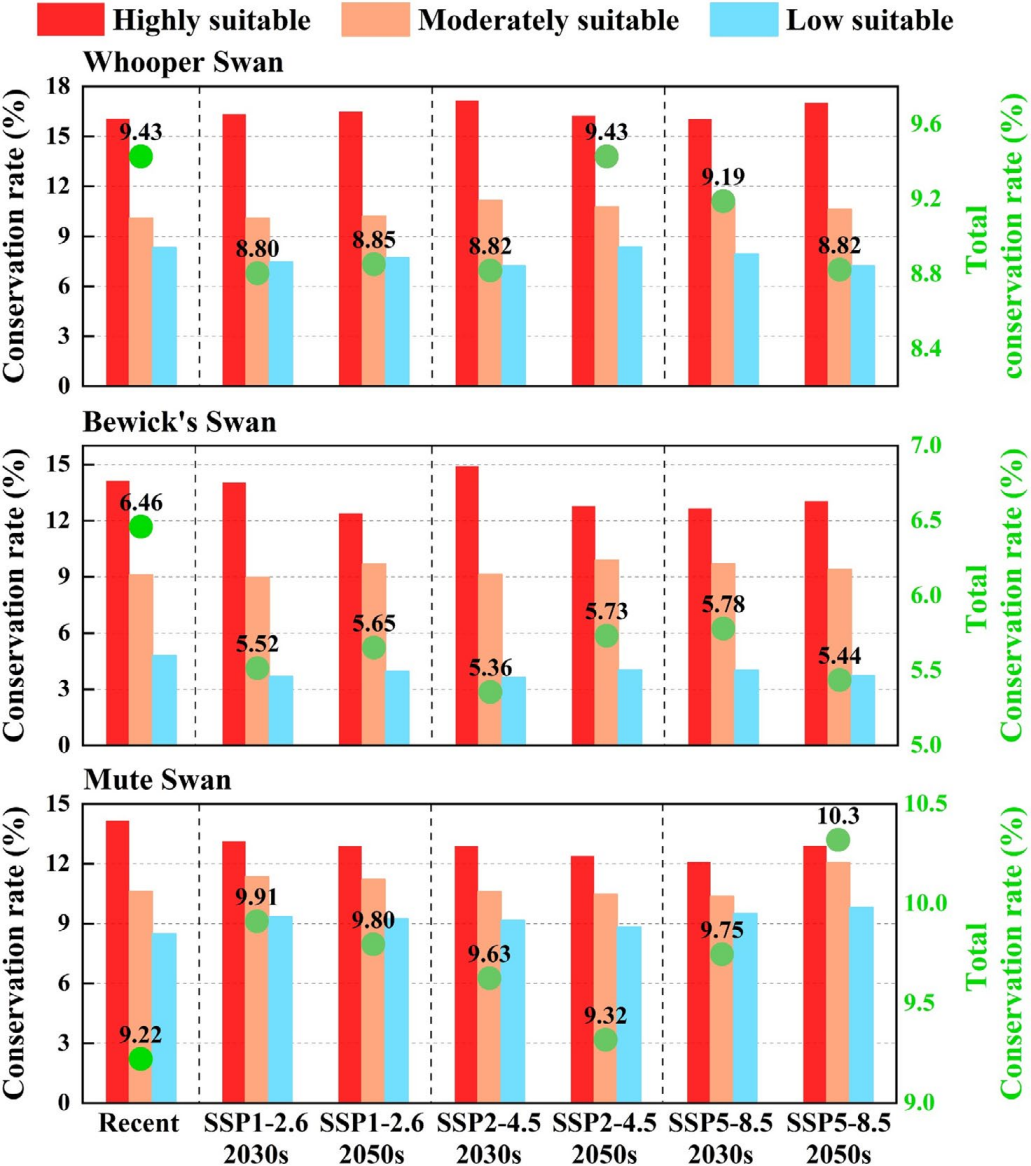
Conservation rates of suitable habitats for the three swan species in China under recent and future climate scenarios. Red, orange, and blue bars represent the conservation rates by suitability class (highly, moderately, and low suitability habitats, respectively), while green dots represent the total conservation rate.

To evaluate the coverage of swan habitats by China's existing protected areas, we overlaid MaxEnt predictions with protected area boundaries and quantified both the total conservation rate and the conservation rate by suitability class for the recent and future periods. In terms of total conservation rate, Whooper Swan remained below the recent level of 9.43% in most future scenarios, except under SSP2–4.5 in the 2050s, where it equaled the recent value. The lowest rate occurred under SSP1–2.6 in the 2030s (8.80%), a decrease of 0.64% compared with the recent period, which corresponded to a loss of about 0.54 × 10^4^ km^2^ of protected habitat. For Bewick's Swan, total conservation rates were consistently lower than the recent value of 6.46% across all scenarios. The lowest rate was 5.63% under SSP2–4.5 in the 2030s, which corresponded to only 3.77 × 10^4^ km^2^ of suitable habitats under protection. In contrast, Mute Swan showed higher total conservation rates than the recent level of 9.22% under all scenarios. The highest rate reached 10.3% under SSP5–8.5 in the 2050s, an increase of 1.08% compared with the recent period, which corresponded to a gain of about 2.74 × 10^4^ km^2^ of protected habitats (Figure 5).

For conservation rate by suitability class, all three species showed a consistent gradient across periods: highly suitable > moderately suitable > low suitable habitats. This indicated that existing protected areas overlap relatively well with highly suitable habitats but insufficiently with moderately and low suitable ones. For Whooper Swan, conservation rates were 16.05%–17.17% for highly suitable habitats, 10.09%–11.16% for moderately suitable habitats, and 7.22%–8.36% for low suitable habitats. For Bewick's Swan, highly suitable habitats had protection rates of 12.38%–14.9%, moderately suitable habitats 8.98%–9.9%, and low suitable habitats below 4.8%. In contrast, Mute Swan showed a more balanced pattern, with conservation rates of 12.11%–14.16% for highly suitable habitats, 10.38%–12.06% for moderately suitable habitats, and 8.5%–9.83% for low suitable habitats (Figure 5).

Based on the main analysis (MTSS), the total conservation rates for each species, scenario, and period were recalculated using the 10% TP and ESS thresholds. The results showed that the differences in total conservation rates among the three thresholds were minimal, with the maximum‐minimum range being only 0.28%–1.46% (Table S7). This indicates that the total conservation rates were not sensitive to threshold selection and were only weakly affected by threshold choice.

## 4 Discussion

### 4.1 Effects of Environmental Variables on Swan Habitats

Understanding species' responses to environmental variables is fundamental to explaining their ecological adaptations and distribution patterns (Yang et al. 2025). Response curves under the MTSS threshold further clarified the ecological ranges of these variables (Figures S4–S6). Based on MaxEnt and Jackknife analyses, this study identified slope (close to 0°), NDVI (0.2–0.8), Dis‐water (< 1 km), and Bio3 (16–30) as shared key variables influencing the suitable habitats of Whooper Swan, Bewick's Swan, and Mute Swan. Collectively, these variables interact to form the core ecological conditions that sustain habitat quality and resource availability (Wang et al. 2022; Wilson et al. 2025). Swans generally tend to select near‐flat areas (slopes close to 0°), which facilitate foraging, resting, and nesting. Such terrain facilitates flexible movement between water and land, thereby reducing energy expenditure. This is particularly important for juveniles, as they require greater protection and energy reserves during their growth process (Liang et al. 2021; Wang et al. 2022). As an important indicator of vegetation cover and ecosystem health, NDVI is closely associated with resource availability and population abundance (Dubinin et al. 2018; Pettorelli et al. 2011). Higher NDVI values typically correspond to greater food resources, nesting sites, and refuges, which directly influence survival during both breeding and wintering periods (Selwood et al. 2009). Dense vegetation can also buffer human disturbance, thereby supporting larger swan populations (Hurlbert 2004). The strong effect of Dis‐water reflects swans' reliance on water–land ecotones, particularly mosaics of water bodies, shallow wetlands, and surrounding vegetation (Pöysä et al. 2013; Yan et al. 2024). Proximity to water enhances foraging efficiency and provides essential stopover resources during migration (Runge et al. 2014). Finally, climatic stability, represented by Bio3, emerged as a common preference across species. All three swans favored Bio3 values between 16 and 30, suggesting a tendency to select habitats with stable temperature regimes. This aligns with previous findings that frequent temperature fluctuations and extreme events increase physiological stress in waterbirds, reducing reproductive success and population stability (Doyle et al. 2020; Kumar et al. 2024). Thus, climatically stable regions may serve as critical refuges for swans under future climate change (Nuijten et al. 2020).

In terms of interspecific differences, the three swan species exhibited distinct variable dependencies, reflecting differentiation in ecological niches and adaptive strategies. Whooper Swan showed high sensitivity to multiple bioclimatic variables, particularly Bio5 (18°C–36°C), Bio9 (−22°C to 28°C), and Bio13 (13–365 mm). The combined influence of these variables suggests that its suitable habitats are shaped by multidimensional climatic conditions, with a particular dependence on warm, humid, and resource‐abundant environments (Holopainen et al. 2024; Jia et al. 2018; Liu et al. 2018). This composite climate–resource dependency may confer greater adaptive capacity and flexibility in habitat selection under varying ecological conditions. In contrast, Bewick's Swan displayed more concentrated dependence on a narrow set of variables, indicating stronger associations with low‐altitude plain wetlands. This reliance indicates limited spatial flexibility and a higher risk of habitat loss or contraction in the future under pressures such as urban expansion or hydrological changes (Yu et al. 2019; Zhang et al. 2021). For Mute Swan, positive responses to Bio12 and Bio19 indicate that its habitat suitability is strongly dependent on precipitation regimes. Precipitation regulates soil moisture and vegetation growth, which directly influence food availability (Liu et al. 2020). In arid and semi‐arid regions, reduced rainfall markedly decreases soil moisture, limiting vegetation recovery and resource availability, thereby exerting pressure on waterbird survival (White et al. 2022). Extreme precipitation events may further disrupt reproduction; for instance, floods during the nesting period can inundate or destroy nests (Debata 2019). Thus, Mute Swan shows strong dependence on stable, moderate precipitation, and its future distribution may be constrained by increasing climatic extremes.

Overall, while the three swan species share common key environmental drivers, they also exhibit distinct climatic adaptation patterns, highlighting interspecific differences in ecological requirements and spatial strategies. These findings suggest that conservation planning should integrate species‐specific habitat requirements and adopt more targeted management and dynamic adjustments.

### 4.2 Adjustment of Habitat Suitability Structure in Swans

Migratory species are particularly sensitive to climate change, and whether they can adapt to ongoing and future climatic shifts remains debated (Van Doren 2022). Traditionally, migratory waterbirds are thought to exhibit strong fidelity to specific habitats throughout their life cycle (van Wijk et al. 2016). This fidelity is rooted in socialized migratory behavior and reliance on familiar habitats, which offer stability and predictable food resources (Franklin et al. 2022). However, climate change can disrupt these patterns, forcing waterbirds to adjust to new temperature and precipitation regimes. Such changes may reduce the suitability of traditional habitats and alter overall distribution patterns (Antão et al. 2022; Linssen et al. 2023; Virkkala and Lehikoinen 2017). Under future climate scenarios, the overall geographic extent of potential suitable habitats for the three swan species is projected to remain largely stable, but their suitability structure shows marked adjustments: low‐suitability areas generally expand, whereas the proportions of medium‐ and high‐suitability habitats remain essentially unchanged (Figure S3 and Figure 4). This expansion is mainly driven by the improvement of suitability levels in currently unsuitable areas, suggesting that some regions previously considered unsuitable may gradually transform into marginal habitats that only meet the minimum ecological requirements. In terms of spatial configuration, suitable habitats of the three swan species typically exhibit a hierarchical pattern of “high‐suitability core—medium‐suitability buffer—low‐suitability periphery.” This pattern reflects an early ecological response of swans to climate change: core high‐quality habitats do not expand significantly, while the proportion of marginal habitats increases, thereby posing potential risks to population viability.

The uncertainty analysis further revealed a consistent pattern across the three swan species: predictions of high‐ and medium‐suitability habitats were generally stable across different GCMs (with mean coefficients of variation ranging from 4% to 6% across species and maximum values not exceeding 11%), whereas low‐suitability habitats showed relatively higher uncertainty, with both mean and maximum values generally exceeding those of the higher suitability classes (mean CV ranging from 6% to 10%, maximum 9%–15%) (Table S6). Overall, the predictions for core habitats appear more robust and can provide a reliable basis for identifying conservation priority areas. By contrast, predictions for marginal habitats are more sensitive to differences among climate models, underscoring the need to account for the uncertainty of their expansion in future research and conservation planning. Long‐term ecological assessments are also essential to determine whether newly emerging low‐suitability areas can sustain populations in the long run, or merely represent transient habitats driven by climate change. These findings highlight the need for dynamic adjustment and optimization of protected‐area networks to accommodate shifting.

### 4.3 Conservation Coverage of Swan Habitats and Optimization Recommendations

At present, substantial knowledge gaps remain in identifying key habitats of migratory species and assessing their conservation status (Zhang, Wei, and Xu 2023). Under climate change, this limitation further highlights the need for systematic evaluations of habitat conservation levels. Overall, China's existing protected areas provide relatively good coverage of the highly suitable habitats of the three swan species, but protection of moderately and low suitable habitats is clearly insufficient (Figure 5). Conservation rates exhibited a consistent gradient of “highly suitable > moderately suitable > low suitable,” suggesting that the current system covers core habitats to some extent, but transitional and marginal zones remain underrepresented. Notably, the total conservation rate of swan habitats under future climate scenarios consistently remained below 11%. Building on these findings, we identified major protection gaps in the potential habitats of the three species, providing a foundation for optimizing regional conservation planning (Erwin 2009; Sun et al. 2020). Habitat gaps were concentrated in northern and central Xinjiang (including the Irtysh River Basin, Ili River Valley, and Bayinbuluke region), the coastal plains around the Yellow Sea and Bohai Bay, and the middle and lower Yangtze River and its tributary plains (e.g., Jiangxi, Hubei, Anhui, Jiangsu). Many of these areas are projected to remain suitable habitats under future scenarios. Although some are already designated as reserves, a significant mismatch persists between reserve boundaries and potential suitable habitats, leaving large areas unprotected. This mismatch arises largely from static planning that neglects the dynamic distributions of migratory birds (Zhang, Zeng, and Namaiti 2023). As a result, when swans move beyond reserve boundaries, they are exposed to higher risks of disturbance (Wilson et al. 2025). With ongoing climate change, relying solely on the existing reserve network is insufficient to secure long‐term population stability.

In this regard, we propose three recommendations: (1) Optimize spatial layout and expand coverage. Based on future habitat projections and the current reserve system, priority should be given to filling spatial gaps in highly suitable but insufficiently protected areas, thereby improving the systematic and comprehensive nature of the network (Chen et al. 2024). (2) Enhance connectivity along migratory pathways (Liz et al. 2022). Moderately and low suitable habitats, although not core areas, play crucial transitional roles during migration, foraging, and dispersal. Strengthening structural connectivity through ecological corridors and wetland buffer zones can help maintain continuity (Dang et al. 2024; He et al. 2024). (3) Increase management flexibility and adaptive capacity. Given regional differences in land use and policy, flexible measures such as community co‐management and temporary protection should be implemented. In areas heavily affected by urban expansion, refined management strategies are needed to mitigate continuous anthropogenic pressures (Duffield et al. 2024; He et al. 2023; Ma et al. 2019). (4) Integrate water resource management and habitat quality into conservation planning. The distribution of swans largely depends on hydrological processes, wetland extent, and food availability, which together with climate determine habitat suitability. Future conservation strategies should incorporate measures such as wetland restoration, sustainable water resource allocation, and habitat quality monitoring into reserve planning, so as to ensure stable foraging and stopover sites for migratory waterbirds and enhance the effectiveness of static protected area networks under climate change (Herse et al. 2025). Together, these measures can improve the adaptability of the protected‐area system and are essential for ensuring the persistence of swan populations under accelerating climate change.

### 4.4 Limitations

Notably, our study has certain limitations. First, when predicting the potential distributions of the three swan species under future climate scenarios, the analysis primarily considered climatic and topographic factors, while some key non‐climatic drivers such as food availability and water resource management were not included. In addition, due to the lack of future projections for NDVI and Human Footprint data, these two variables were excluded from the modeling under future scenarios. As a result, our evaluation of habitat suitability is more focused on the effects of climatic factors (Lee et al. 2023; Zhu et al. 2022). Second, breeding and wintering grounds were not distinguished, and the models were built from the perspective of overall distribution. This may limit the understanding of seasonal ecological requirements and distribution dynamics of swans (Wang et al. 2025). Third, the conservation of migratory waterbirds depends not only on the spatial coverage of protected areas but also on management effectiveness, wetland protection, and habitat scale, which are all critical for maintaining swan populations. Given that swans are long‐distance migrants highly dependent on a dynamic network of breeding, stopover, and wintering sites, future studies should further incorporate dynamic environmental factors, address seasonal ecological requirements, and place greater emphasis on the connectivity and integrity of flyway‐scale conservation.

## 5 Conclusion

This study examined the distribution and conservation of suitable habitats for Whooper Swan, Bewick's Swan, and Mute Swan in China under recent and future climate scenarios. The main findings are as follows: (1) the MaxEnt model showed high predictive accuracy for all three species; (2) in the recent period, swans exhibited common responses to environmental variables, preferring near‐flat areas with good vegetation cover, proximity to water, and stable climatic conditions; (3) under future climate scenarios, the overall spatial ranges of suitable habitats remained relatively stable, but the suitability structure shifted, with low‐suitability habitats generally expanding; and (4) existing protected areas provided relatively good coverage of highly suitable habitats but insufficient coverage of moderately and low‐suitable habitats, with total conservation rates for all three species consistently below 11%, indicating substantial protection gaps. These findings enhance our understanding of swan habitat distribution and conservation under climate change and provide a scientific basis for optimizing the planning and management of migratory bird habitat conservation networks in China.

## Author Contributions

**Ke Zhang:** conceptualization (equal), data curation (equal), formal analysis (equal), investigation (equal), methodology (equal), software (equal), visualization (equal), writing – original draft (equal). **Jun Lin:** funding acquisition (equal), investigation (equal), writing – review and editing (equal). **Jianghua Zheng:** data curation (equal), funding acquisition (equal), methodology (equal), supervision (equal), writing – review and editing (equal). **Xuan Li:** investigation (equal), writing – review and editing (equal). **Li Xu:** methodology (equal), software (equal), writing – review and editing (equal). **Liang Liu:** writing – review and editing (equal). **Xuan Liu:** writing – review and editing (equal). **Xi Jin:** writing – review and editing (equal). **Rong Fu:** methodology (equal), software (equal), writing – review and editing (equal). **Xinwei Wang:** writing – review and editing (equal). **Yunzhi Sang:** writing – review and editing (equal). **Xiaoyu Guo:** writing – review and editing (equal).

## Conflicts of Interest

The authors declare no conflicts of interest.

## Supporting information


**Appendix S1:** ece372238‐sup‐0001‐AppendixS1.docx.

## Data Availability

The data and materials that support the findings of this study are provided in the Supporting Information.
