# Supplementary material for: Evaluating Climate Change Effects on Swan Habitats Within China: Adaptive Strategies for Sustainable Conservation
Source: Ecol Evol. 2025 Oct 1;15(10):e72238. doi: 10.1002/ece3.72238 (PMC12486193; doi:10.1002/ece3.72238)
Supplement: Supplementary file 1 — Appendix S1: ece372238‐sup‐0001‐AppendixS1.docx. [file ECE3-15-e72238-s001.docx]

**Supplementary Materials for**

***Evaluating Climate Change Effects on Swan Habitats within China: Adaptive Strategies for Sustainable Conservation***

This file includes:

Tables S1 to S7

Figures S1 to S6

**Table S1.** Final occurrence records of *Cygnus cygnus*, *Cygnus columbianus*, and *Cygnus olor* after data filtering.

| **species** | **Information** | **Information** | **Information** | **Information** | **Information** |
| --- | --- | --- | --- | --- | --- |
| ***Cygnus cygnus*** | (119.18E,37.77N) | (122.46E,37.14N) | (87.44E,47.59N) | (123.39E,41.29N) | (99.86E,37.03N) |
|  | (115.83E,40.40N) | (117.39E,38.74N) | (87.33E,47.50N) | (130.61E,42.60N) | (99.79E,36.94N) |
|  | (116.02E,29.19N) | (117.39E,38.71N) | (87.29E,47.60N) | (123.66E,40.15N) | (100.68E,36.82N) |
|  | (116.27E,40.00N) | (116.39E,29.11N) | (87.10E,47.57N) | (119.50E,39.85N) | (100.70E,36.70N) |
|  | (116.30E,40.01N) | (116.56E,29.16N) | (87.51E,47.53N) | (119.26E,39.59N) | (115.63E,40.31N) |
|  | (111.15E,34.81N) | (116.43E,29.08N) | (86.73E,47.79N) | (119.02E,37.71N) | (111.23E,34.79N) |
|  | (111.14E,34.82N) | (116.37E,29.09N) | (86.51E,44.57N) | (118.74E,37.59N) | (111.20E,34.80N) |
|  | (116.59E,40.31N) | (116.41E,29.14N) | (82.34E,44.91N) | (118.79E,37.35N) | (119.28E,43.26N) |
|  | (115.85E,40.41N) | (121.58E,40.93N) | (82.25E,44.77N) | (118.99E,37.82N) | (122.94E,42.36N) |
|  | (100.72E,36.56N) | (112.66E,34.85N) | (82.80E,45.07N) | (118.77E,38.07N) | (119.48E,43.27N) |
|  | (111.13E,34.78N) | (117.42E,38.80N) | (81.63E,45.00N) | (118.72E,38.02N) | (122.60E,37.36N) |
|  | (116.97E,36.74N) | (114.86E,35.18N) | (81.40E,45.03N) | (118.08E,37.99N) | (122.56E,37.35N) |
|  | (118.97E,37.74N) | (100.16E,36.19N) | (86.12E,44.39N) | (122.56E,37.36N) | (122.57E,37.34N) |
|  | (120.58E,33.50N) | (116.98E,40.49N) | (81.40E,44.97N) | (119.12E,37.72N) | (122.59E,37.34N) |
|  | (115.83E,40.38N) | (119.17E,37.74N) | (82.55E,44.85N) | (120.11E,36.21N) | (115.83E,40.41N) |
|  | (118.83E,32.05N) | (101.54E,36.11N) | (81.27E,44.59N) | (120.19E,35.92N) | (115.86E,40.44N) |
|  | (116.57E,40.31N) | (119.08E,37.81N) | (85.95E,44.81N) | (120.20E,35.93N) | (115.81E,40.42N) |
|  | (115.98E,28.69N) | (120.64E,33.48N) | (85.89E,45.79N) | (119.49E,35.64N) | (115.77E,40.38N) |
|  | (117.52E,39.28N) | (121.98E,30.90N) | (87.55E,47.43N) | (119.44E,35.31N) | (115.66E,40.32N) |
|  | (121.96E,31.50N) | (118.77E,37.40N) | (87.41E,47.64N) | (120.55E,33.60N) | (115.74E,40.35N) |
|  | (119.71E,48.93N) | (84.29E,42.85N) | (85.56E,48.00N) | (118.80E,32.07N) | (115.61E,40.29N) |
|  | (101.89E,36.57N) | (81.22E,44.53N) | (87.07E,47.78N) | (117.30E,31.58N) | (115.60E,40.27N) |
|  | (119.08E,37.78N) | (81.13E,44.72N) | (87.10E,47.30N) | (117.03E,30.83N) | (120.50E,41.59N) |
|  | (115.98E,29.18N) | (81.29E,44.54N) | (87.28E,47.03N) | (117.14E,30.87N) | (120.48E,41.58N) |
|  | (116.31E,28.77N) | (88.19E,47.70N) | (86.69E,48.01N) | (117.53E,30.65N) | (130.57E,42.62N) |
|  | (114.88E,43.42N) | (83.01E,46.47N) | (87.41E,47.47N) | (115.74E,29.71N) | (115.85E,40.40N) |
|  | (116.97E,40.54N) | (86.11E,44.17N) | (80.92E,44.99N) | (121.95E,31.55N) | (121.98E,31.51N) |
|  | (115.81E,40.38N) | (86.00E,44.43N) | (85.68E,44.87N) | (121.92E,31.47N) | (121.96E,31.53N) |
|  | (115.78E,40.37N) | (87.73E,44.20N) | (85.23E,45.43N) | (120.31E,31.35N) | (121.95E,31.52N) |
|  | (116.18E,40.10N) | (87.67E,43.62N) | (85.56E,46.08N) | (121.95E,30.99N) | (121.97E,31.52N) |
|  | (116.33E,28.90N) | (81.02E,44.65N) | (85.64E,46.10N) | (116.19E,29.00N) | (121.96E,31.51N) |
|  | (117.45E,38.48N) | (87.03E,41.99N) | (86.25E,46.86N) | (116.32E,28.71N) | (100.47E,38.98N) |
|  | (116.43E,28.87N) | (87.89E,44.25N) | (86.37E,44.37N) | (117.35E,39.05N) | (87.11E,43.89N) |
|  | (116.49E,28.85N) | (83.31E,43.51N) | (86.15E,46.74N) | (117.15E,39.02N) | (86.24E,44.42N) |
|  | (115.97E,29.14N) | (84.61E,44.77N) | (83.94E,46.68N) | (117.19E,38.92N) | (82.96E,46.92N) |
|  | (118.96E,37.80N) | (87.92E,43.50N) | (85.71E,45.74N) | (116.95E,39.05N) | (82.99E,46.76N) |
|  | (117.42E,38.79N) | (86.14E,44.44N) | (86.16E,45.01N) | (117.29E,38.74N) | (87.01E,47.61N) |
|  | (96.92E,37.25N) | (88.26E,43.36N) | (83.81E,46.59N) | (116.95E,43.26N) | (86.87E,47.69N) |
|  | (116.64E,43.28N) | (81.10E,44.52N) | (86.27E,47.85N) | (116.51E,43.24N) | (86.64E,47.84N) |
|  | (115.63E,40.32N) | (86.24E,41.59N) | (85.85E,44.77N) | (115.01E,43.46N) | (85.88E,47.96N) |
|  | (118.69E,37.57N) | (86.65E,41.89N) | (85.83E,44.82N) | (111.10E,34.75N) | (85.70E,47.97N) |
|  | (117.35E,38.74N) | (87.16E,42.04N) | (85.92E,44.55N) | (111.12E,34.77N) | (82.03E,44.96N) |
|  | (119.26E,48.86N) | (87.09E,42.07N) | (86.15E,44.50N) | (115.46E,40.37N) | (82.33E,44.67N) |
|  | (117.03E,40.51N) | (86.66E,42.24N) | (85.78E,41.59N) | (116.48E,39.95N) | (81.33E,44.91N) |
|  | (116.59E,34.49N) | (84.54E,43.04N) | (86.01E,41.39N) | (116.21E,39.82N) | (81.81E,44.95N) |
|  | (117.09E,30.75N) | (86.30E,41.82N) | (85.97E,41.17N) | (116.24E,39.81N) | (82.28E,44.85N) |
|  | (111.21E,34.79N) | (86.18E,41.70N) | (85.79E,41.45N) | (116.46E,39.77N) | (83.10E,46.68N) |
|  | (115.82E,40.40N) | (86.26E,42.20N) | (86.05E,41.07N) | (116.61E,40.33N) | (83.27E,46.50N) |
|  | (116.32E,28.76N) | (85.20E,42.97N) | (81.18E,44.61N) | (116.61E,40.32N) | (82.82E,46.51N) |
|  | (118.96E,37.70N) | (84.81E,43.04N) | (80.58E,43.99N) | (117.07E,40.53N) | (82.41E,44.62N) |
|  | (121.86E,41.00N) | (86.11E,41.28N) | (82.89E,44.90N) | (117.13E,40.59N) | (82.48E,44.64N) |
|  | (112.39E,37.61N) | (84.39E,42.82N) | (122.58E,37.36N) | (117.09E,40.56N) | (82.26E,44.63N) |
|  | (99.90E,36.98N) | (84.45E,42.74N) | (122.56E,37.29N) | (116.92E,40.55N) | (84.22E,46.90N) |
|  | (114.16E,30.75N) | (85.97E,42.23N) | (122.51E,37.16N) | (116.66E,39.91N) | (86.10E,47.90N) |
|  | (116.36E,28.95N) | (86.43E,41.92N) | (122.45E,37.12N) | (116.31E,40.13N) | (87.93E,47.77N) |
|  | (114.05E,34.94N) | (86.45E,42.10N) | (122.41E,36.91N) | (116.33E,40.13N) | (87.98E,47.73N) |
|  | (117.53E,38.64N) | (86.42E,41.80N) | (122.12E,37.36N) | (116.30E,40.14N) | (87.92E,47.83N) |
|  | (112.54E,34.88N) | (86.32E,42.27N) | (120.44E,37.00N) | (116.20E,40.11N) | (81.72E,44.96N) |
|  | (115.90E,29.15N) | (85.59E,42.29N) | (120.45E,36.94N) | (116.62E,40.36N) | (82.91E,44.81N) |
|  | (116.27E,39.99N) | (84.19E,42.85N) | (120.77E,36.61N) | (116.75E,40.30N) | (82.69E,44.87N) |
|  | (122.24E,41.26N) | (84.37E,43.08N) | (86.80E,47.79N) | (116.26E,40.26N) | (85.85E,44.70N) |
|  | (120.55E,33.56N) | (84.11E,42.91N) | (86.82E,47.85N) | (116.26E,40.19N) | (86.05E,44.80N) |
|  | (120.53E,33.65N) | (84.23E,42.76N) | (88.25E,47.63N) | (116.45E,40.24N) | (87.54E,47.27N) |
|  | (115.88E,40.47N) | (84.30E,42.80N) | (88.19E,47.64N) | (116.52E,40.07N) | (100.46E,38.97N) |
|  | (110.94E,34.70N) | (86.04E,41.28N) | (88.04E,47.66N) | (116.02E,39.73N) | (86.10E,42.27N) |
|  | (111.71E,40.77N) | (86.13E,41.33N) | (87.77E,47.62N) | (116.03E,39.69N) | (86.77E,42.14N) |
|  | (123.16E,41.33N) | (84.08E,42.87N) | (87.36E,47.33N) | (114.82E,40.68N) | (85.96E,41.75N) |
|  | (123.39E,41.30N) | (83.99E,42.74N) | (87.08E,48.86N) | (114.96E,40.63N) | (86.24E,41.62N) |
|  | (120.41E,36.95N) | (84.10E,42.81N) | (87.01E,48.75N) | (115.73E,39.56N) | (117.53E,48.36N) |
|  | (116.84E,43.30N) | (84.13E,42.75N) | (87.11E,48.12N) | (115.78E,39.52N) | (117.39E,48.28N) |
|  | (108.87E,40.93N) | (85.53E,41.74N) | (87.13E,48.16N) | (111.40E,40.68N) | (118.91E,48.80N) |
|  | (100.45E,37.05N) | (85.63E,41.67N) | (86.82E,48.16N) | (113.64E,39.71N) | (119.06E,48.81N) |
|  | (117.07E,48.75N) | (86.05E,41.67N) | (86.35E,48.09N) | (113.65E,39.72N) | (124.12E,50.40N) |
|  | (117.41E,31.70N) | (82.66E,41.07N) | (86.25E,47.92N) | (109.89E,39.10N) | (124.23E,47.20N) |
|  | (120.91E,41.76N) | (84.21E,42.80N) | (86.43E,47.87N) | (108.95E,39.26N) | (123.04E,46.26N) |
|  | (111.15E,34.78N) | (86.81E,42.03N) | (87.85E,47.61N) | (106.76E,39.61N) | (122.25E,45.08N) |
|  | (116.67E,43.38N) | (86.58E,42.23N) | (87.52E,47.74N) | (106.38E,38.81N) | (122.50E,44.96N) |
|  | (108.90E,34.18N) | (84.74E,43.03N) | (88.48E,47.24N) | (106.33E,38.80N) | (120.93E,44.02N) |
|  | (117.50E,38.52N) | (84.64E,43.07N) | (88.11E,47.15N) | (108.87E,41.09N) | (120.24E,44.33N) |
|  | (122.35E,45.02N) | (84.94E,43.02N) | (87.91E,46.53N) | (108.90E,41.01N) | (119.11E,43.20N) |
|  | (115.59E,40.29N) | (85.01E,43.02N) | (90.61E,46.40N) | (108.81E,40.88N) | (119.78E,42.42N) |
|  | (123.70E,45.91N) | (85.05E,42.98N) | (87.55E,47.64N) | (108.73E,40.82N) | (123.31E,42.75N) |
|  | (123.65E,45.91N) | (86.42E,41.06N) | (86.38E,46.89N) | (109.51E,38.07N) | (123.25E,42.69N) |
|  | (120.61E,30.38N) | (83.42E,41.63N) | (86.13E,46.85N) | (109.44E,38.06N) | (123.21E,42.72N) |
|  | (99.10E,36.72N) | (82.68E,40.99N) | (86.39E,46.34N) | (112.53E,37.91N) | (123.28E,42.71N) |
|  | (116.22E,28.89N) | (83.25E,41.01N) | (86.08E,45.89N) | (112.40E,37.62N) | (123.30E,42.72N) |
|  | (115.60E,40.32N) | (83.60E,41.26N) | (85.97E,46.66N) | (115.63E,37.65N) | (122.93E,42.37N) |
|  | (116.08E,28.81N) | (83.95E,41.79N) | (85.35E,46.15N) | (115.58E,37.58N) | (123.59E,41.82N) |
|  | (117.36E,38.78N) | (84.33E,41.46N) | (83.20E,47.00N) | (118.34E,39.19N) | (123.37E,41.75N) |
|  | (120.52E,33.59N) | (83.95E,41.17N) | (83.77E,46.52N) | (118.38E,39.18N) | (119.75E,41.07N) |
|  | (116.52E,28.92N) | (84.40E,41.19N) | (81.11E,44.99N) | (115.59E,37.62N) | (121.14E,41.09N) |
|  | (115.61E,40.30N) | (84.29E,43.05N) | (82.65E,45.08N) | (117.50E,38.49N) | (121.24E,40.88N) |
|  | (115.55E,40.32N) | (83.69E,41.15N) | (83.08E,44.89N) | (117.48E,38.50N) | (121.26E,40.99N) |
|  | (116.55E,40.04N) | (84.81E,41.18N) | (81.32E,45.03N) | (114.71E,41.35N) | (121.56E,40.87N) |
|  | (111.15E,34.83N) | (83.08E,40.99N) | (82.02E,44.89N) | (114.74E,41.35N) | (123.20E,41.33N) |
|  | (111.27E,34.80N) | (86.36E,42.23N) | (82.62E,45.15N) | (114.72E,41.34N) | (99.74E,39.41N) |
|  | (122.34E,45.04N) | (85.44E,42.90N) | (82.67E,44.78N) | (113.58E,39.93N) | (102.33E,34.21N) |
|  | (115.57E,40.29N) | (85.12E,42.98N) | (81.03E,44.57N) | (113.57E,39.92N) | (102.35E,34.22N) |
|  | (115.69E,40.36N) | (85.32E,42.94N) | (81.21E,44.68N) | (113.54E,39.91N) | (102.34E,34.24N) |
|  | (114.22E,34.91N) | (83.55E,43.47N) | (82.70E,43.52N) | (113.61E,39.94N) | (102.38E,34.22N) |
|  | (117.68E,39.13N) | (86.92E,41.88N) | (81.12E,43.00N) | (116.97E,36.76N) | (102.83E,33.92N) |
|  | (117.79E,48.06N) | (85.08E,43.03N) | (81.17E,42.94N) | (116.81E,36.65N) | (102.30E,34.30N) |
|  | (122.31E,45.00N) | (86.43E,41.43N) | (83.84E,43.42N) | (116.82E,36.68N) | (102.46E,35.14N) |
|  | (122.39E,44.98N) | (86.04E,41.18N) | (84.82E,42.99N) | (116.60E,34.50N) | (102.50E,35.31N) |
|  | (115.94E,29.09N) | (84.01E,42.86N) | (85.60E,42.85N) | (116.57E,34.50N) | (104.60E,35.61N) |
|  | (96.89E,37.30N) | (82.86E,40.98N) | (85.62E,42.81N) | (113.71E,34.82N) | (103.37E,36.12N) |
|  | (120.54E,33.52N) | (83.64E,41.09N) | (84.13E,43.00N) | (113.67E,34.92N) | (102.33E,35.87N) |
|  | (119.02E,37.83N) | (83.43E,41.00N) | (84.22E,43.05N) | (112.64E,34.85N) | (101.94E,36.06N) |
|  | (100.50E,37.00N) | (82.54E,41.10N) | (83.98E,42.82N) | (111.08E,35.03N) | (101.60E,36.14N) |
|  | (122.96E,42.37N) | (85.50E,41.03N) | (83.85E,42.86N) | (111.00E,34.99N) | (101.53E,36.10N) |
|  | (116.69E,30.03N) | (87.26E,40.75N) | (86.24E,44.72N) | (110.89E,34.93N) | (100.72E,36.19N) |
|  | (117.15E,48.59N) | (86.59E,41.03N) | (86.04E,44.60N) | (110.59E,34.61N) | (100.73E,36.16N) |
|  | (120.70E,31.27N) | (86.25E,41.17N) | (86.09E,44.12N) | (109.01E,34.43N) | (100.73E,36.06N) |
|  | (117.44E,48.97N) | (86.50E,41.94N) | (86.15E,41.76N) | (112.83E,34.14N) | (100.88E,36.14N) |
|  | (109.01E,34.42N) | (86.60E,41.84N) | (87.34E,41.97N) | (109.48E,24.33N) | (100.41E,35.79N) |
|  | (118.79E,32.08N) | (87.16E,41.92N) | (86.70E,41.05N) | (104.22E,26.84N) | (100.10E,36.19N) |
|  | (119.34E,48.90N) | (86.76E,41.91N) | (86.71E,40.96N) | (104.23E,26.83N) | (100.12E,36.19N) |
|  | (119.33E,48.95N) | (86.86E,42.09N) | (85.79E,41.18N) | (104.27E,26.85N) | (99.74E,37.03N) |
|  | (106.38E,38.83N) | (85.86E,41.76N) | (84.09E,41.12N) | (104.14E,30.74N) | (100.25E,36.74N) |
|  | (119.01E,37.79N) | (85.91E,41.40N) | (84.02E,42.81N) | (102.83E,33.91N) | (100.18E,36.98N) |
|  | (119.04E,37.75N) | (85.90E,44.45N) | (84.15E,42.79N) | (102.86E,33.78N) | (100.00E,37.17N) |
|  | (119.07E,37.74N) | (86.17E,45.86N) | (86.62E,41.97N) | (102.83E,33.81N) | (100.21E,37.23N) |
|  | (101.03E,36.87N) | (87.22E,47.19N) | (85.61E,41.72N) | (102.46E,33.48N) | (101.34E,37.60N) |
|  | (117.58E,48.42N) | (87.90E,47.48N) | (84.84E,41.41N) | (102.54E,33.99N) | (101.33E,37.61N) |
|  | (111.39E,40.67N) | (88.89E,46.38N) | (85.51E,42.33N) | (102.46E,34.02N) | (101.42E,37.46N) |
|  | (100.50E,36.58N) | (87.99E,44.20N) | (85.78E,42.23N) | (102.32E,34.24N) | (97.13E,37.17N) |
|  | (96.97E,37.12N) | (99.76E,39.41N) |  |  |  |
| ***Cygnus columbianus*** | (116.02E,29.19N) | (116.32E,28.91N) | (116.33E,28.92N) | (116.93E,40.48N) | (120.57E,33.55N) |
|  | (113.00E,29.35N) | (119.61E,30.90N) | (116.61E,40.31N) | (116.90E,40.51N) | (120.51E,33.61N) |
|  | (115.98E,29.13N) | (116.07E,29.87N) | (116.53E,29.84N) | (117.13E,40.59N) | (116.29E,35.10N) |
|  | (117.42E,38.79N) | (119.55E,31.58N) | (116.30E,31.42N) | (115.60E,40.26N) | (114.58E,31.29N) |
|  | (115.97E,29.14N) | (112.55E,32.99N) | (120.44E,30.36N) | (115.81E,40.42N) | (114.58E,31.29N) |
|  | (116.38E,28.82N) | (117.04E,30.84N) | (120.38E,31.24N) | (115.79E,40.39N) | (103.66E,27.24N) |
|  | (116.04E,29.14N) | (116.01E,29.18N) | (117.50E,38.52N) | (115.84E,40.41N) | (103.65E,27.24N) |
|  | (116.00E,29.19N) | (120.67E,31.36N) | (116.04E,29.20N) | (115.84E,40.44N) | (121.94E,31.47N) |
|  | (116.43E,28.87N) | (114.23E,30.12N) | (116.44E,40.23N) | (115.83E,40.40N) | (120.45E,31.15N) |
|  | (116.82E,36.68N) | (114.22E,30.13N) | (112.93E,29.36N) | (115.78E,40.36N) | (120.53E,31.13N) |
|  | (117.16E,36.51N) | (115.87E,29.09N) | (116.83E,30.26N) | (115.60E,40.28N) | (120.58E,31.10N) |
|  | (120.87E,30.21N) | (115.86E,29.09N) | (115.76E,41.78N) | (115.62E,40.30N) | (120.58E,31.09N) |
|  | (116.47E,28.92N) | (117.42E,31.73N) | (115.72E,41.71N) | (115.57E,40.31N) | (120.56E,31.06N) |
|  | (114.15E,30.76N) | (117.40E,30.60N) | (116.28E,29.13N) | (115.63E,40.32N) | (120.52E,31.04N) |
|  | (116.47E,28.84N) | (120.61E,30.40N) | (116.30E,28.79N) | (115.46E,40.36N) | (120.45E,31.22N) |
|  | (118.83E,31.46N) | (115.84E,29.97N) | (115.73E,29.71N) | (115.45E,40.37N) | (120.37E,31.21N) |
|  | (116.49E,28.91N) | (118.86E,31.46N) | (122.45E,44.99N) | (115.48E,40.37N) | (120.37E,31.23N) |
|  | (115.90E,29.10N) | (116.27E,28.91N) | (116.32E,28.89N) | (116.16E,40.64N) | (120.39E,31.37N) |
|  | (116.31E,28.75N) | (119.76E,29.10N) | (116.32E,28.88N) | (116.69E,28.69N) | (120.38E,31.37N) |
|  | (116.40E,28.84N) | (117.76E,31.64N) | (120.94E,30.18N) | (115.81E,29.02N) | (120.59E,31.41N) |
|  | (116.32E,28.94N) | (122.34E,45.02N) | (111.82E,30.11N) | (116.66E,29.14N) | (120.78E,31.40N) |
|  | (112.80E,29.51N) | (117.04E,30.38N) | (121.35E,30.30N) | (116.11E,29.36N) | (121.63E,31.68N) |
|  | (116.43E,28.82N) | (120.51E,31.03N) | (116.23E,29.90N) | (116.18E,28.55N) | (120.73E,30.50N) |
|  | (120.90E,30.22N) | (112.39E,37.61N) | (115.96E,29.10N) | (116.15E,28.50N) | (120.61E,30.38N) |
|  | (116.45E,28.91N) | (104.33E,30.61N) | (116.34E,28.92N) | (115.98E,28.69N) | (120.75E,30.35N) |
|  | (116.47E,28.83N) | (116.50E,28.91N) | (116.21E,28.88N) | (115.85E,28.72N) | (120.11E,30.10N) |
|  | (120.08E,30.27N) | (115.59E,37.57N) | (120.34E,30.27N) | (116.06E,29.22N) | (119.75E,30.24N) |
|  | (118.91E,31.46N) | (118.93E,37.75N) | (116.11E,29.06N) | (116.07E,29.21N) | (121.68E,30.00N) |
|  | (118.02E,24.54N) | (120.12E,30.18N) | (115.95E,29.12N) | (114.21E,30.09N) | (121.16E,30.32N) |
|  | (100.45E,38.97N) | (117.14E,39.04N) | (116.39E,28.83N) | (113.82E,30.31N) | (119.59E,30.83N) |
|  | (116.47E,28.82N) | (118.14E,32.98N) | (116.41E,28.99N) | (113.86E,30.35N) | (119.47E,30.62N) |
|  | (118.89E,37.64N) | (121.94E,31.52N) | (116.68E,30.24N) | (113.82E,30.35N) | (119.46E,30.53N) |
|  | (116.60E,28.89N) | (119.57E,31.60N) | (113.02E,29.34N) | (113.88E,30.34N) | (119.41E,29.98N) |
|  | (116.60E,28.87N) | (119.97E,29.19N) | (115.78E,29.71N) | (114.37E,30.66N) | (119.37E,30.05N) |
|  | (116.40E,28.85N) | (117.02E,32.97N) | (116.28E,28.69N) | (114.11E,30.50N) | (119.12E,30.30N) |
|  | (113.85E,30.30N) | (100.45E,38.98N) | (115.69E,29.78N) | (114.34E,30.44N) | (119.82E,29.13N) |
|  | (120.89E,30.21N) | (114.78E,30.28N) | (115.84E,29.98N) | (114.42E,30.52N) | (119.96E,29.17N) |
|  | (119.75E,30.25N) | (120.15E,30.24N) | (114.51E,30.81N) | (114.39E,30.56N) | (119.64E,29.05N) |
|  | (121.92E,31.50N) | (120.55E,33.56N) | (112.71E,34.83N) | (114.50E,30.58N) | (119.15E,29.04N) |
|  | (119.62E,26.03N) | (114.57E,38.11N) | (115.70E,29.79N) | (114.17E,30.80N) | (118.81E,28.88N) |
|  | (117.51E,31.29N) | (120.69E,31.64N) | (116.39E,28.80N) | (114.13E,30.78N) | (118.74E,28.84N) |
|  | (117.43E,31.70N) | (116.72E,30.26N) | (116.00E,29.90N) | (114.21E,30.69N) | (118.42E,28.96N) |
|  | (119.16E,29.05N) | (123.60E,41.82N) | (116.24E,29.88N) | (114.59E,30.25N) | (120.72E,29.07N) |
|  | (120.57E,31.37N) | (116.51E,30.04N) | (114.17E,30.84N) | (116.24E,40.27N) | (121.05E,28.59N) |
|  | (115.74E,29.71N) | (119.49E,31.57N) | (116.54E,28.91N) | (116.02E,39.73N) | (119.49E,28.44N) |
|  | (117.63E,30.93N) | (116.43E,28.90N) | (115.99E,29.18N) | (116.07E,39.79N) | (119.49E,28.44N) |
|  | (111.63E,40.74N) | (112.80E,29.50N) | (116.08E,28.81N) | (117.24E,39.45N) | (119.44E,28.47N) |
|  | (121.96E,31.50N) | (116.53E,30.00N) | (117.50E,39.16N) | (117.60E,39.30N) | (119.54E,28.11N) |
|  | (113.05E,38.59N) | (116.68E,40.17N) | (116.99E,30.30N) | (117.51E,39.29N) | (119.63E,26.69N) |
|  | (117.40E,31.72N) | (119.80E,28.36N) | (116.52E,28.92N) | (117.55E,38.90N) | (119.65E,26.02N) |
|  | (120.14E,30.15N) | (116.19E,29.24N) | (116.25E,29.24N) | (117.50E,38.64N) | (119.37E,25.95N) |
|  | (120.60E,31.11N) | (115.92E,29.65N) | (116.03E,39.69N) | (117.21E,38.76N) | (118.12E,24.45N) |
|  | (115.97E,29.18N) | (120.74E,31.61N) | (113.32E,29.89N) | (101.56E,36.12N) | (115.67E,29.80N) |
|  | (113.85E,30.31N) | (120.80E,31.90N) | (116.03E,29.23N) | (100.37E,36.82N) | (115.22E,29.86N) |
|  | (117.11E,30.38N) | (116.20E,32.25N) | (115.71E,29.77N) | (109.72E,38.34N) | (115.77E,29.69N) |
|  | (121.96E,31.51N) | (116.33E,31.46N) | (116.00E,29.14N) | (111.81E,40.77N) | (116.02E,29.74N) |
|  | (121.94E,31.50N) | (112.80E,29.47N) | (117.68E,39.13N) | (115.81E,41.65N) | (117.53E,30.66N) |
|  | (120.80E,31.24N) | (120.45E,31.21N) | (117.15E,30.88N) | (115.01E,43.45N) | (117.34E,31.21N) |
|  | (116.36E,28.95N) | (119.36E,25.95N) | (116.34E,29.11N) | (113.51E,39.90N) | (117.19E,31.87N) |
|  | (116.31E,28.77N) | (117.57E,31.43N) | (116.42E,28.88N) | (112.51E,39.32N) | (117.15E,31.81N) |
|  | (119.91E,30.26N) | (115.90E,29.28N) | (113.55E,39.92N) | (113.04E,38.58N) | (116.35E,31.61N) |
|  | (115.97E,29.12N) | (116.26E,40.19N) | (116.63E,30.28N) | (112.53E,37.90N) | (117.11E,32.16N) |
|  | (119.08E,37.78N) | (116.44E,28.83N) | (116.59E,40.31N) | (112.50E,37.77N) | (117.53E,32.26N) |
|  | (120.29E,29.74N) | (116.26E,29.91N) | (115.95E,29.13N) | (111.58E,35.65N) | (117.13E,32.57N) |
|  | (120.92E,30.22N) | (116.69E,40.25N) | (112.81E,29.51N) | (111.08E,34.75N) | (116.23E,32.58N) |
|  | (117.98E,24.55N) | (118.68E,24.79N) | (119.61E,26.04N) | (111.13E,34.79N) | (118.78E,32.08N) |
|  | (120.77E,31.41N) | (115.76E,29.71N) | (115.98E,29.20N) | (111.15E,34.82N) | (118.80E,32.07N) |
|  | (117.39E,31.18N) | (115.88E,28.69N) | (119.63E,26.02N) | (110.24E,34.84N) | (110.28E,35.02N) |
|  | (120.81E,31.39N) | (117.03E,30.33N) | (116.39E,28.93N) | (114.28E,38.25N) | (110.24E,34.96N) |
|  | (119.93E,30.34N) | (118.22E,33.25N) | (116.40E,28.76N) | (114.53E,38.13N) | (109.92E,39.08N) |
|  | (118.66E,32.01N) | (118.23E,33.29N) | (112.80E,29.48N) | (115.60E,37.60N) | (113.25E,29.81N) |
|  | (116.34E,28.80N) | (118.22E,33.23N) | (115.85E,29.66N) | (115.62E,37.64N) | (113.29E,29.73N) |
|  | (115.99E,29.13N) | (118.34E,33.23N) | (112.82E,29.51N) | (115.54E,38.88N) | (114.63E,30.14N) |
|  | (116.31E,28.92N) | (120.48E,33.76N) | (115.93E,29.17N) | (122.45E,37.13N) | (114.55E,30.22N) |
|  | (116.28E,31.42N) | (117.39E,38.72N) | (116.19E,29.21N) | (120.18E,35.92N) | (114.48E,30.24N) |
|  | (117.02E,30.34N) | (118.52E,31.82N) | (116.34E,29.03N) | (119.49E,35.64N) | (120.86E,33.10N) |
|  | (115.95E,29.08N) | (116.02E,29.16N) | (114.30E,30.59N) | (119.36E,35.42N) | (120.87E,33.05N) |
|  | (117.78E,30.70N) | (118.97E,31.46N) | (117.85E,29.20N) | (119.44E,35.31N) | (120.90E,33.02N) |
|  | (115.93E,29.18N) | (116.18E,29.15N) | (119.02E,37.83N) | (116.80E,36.66N) | (120.85E,33.03N) |
|  | (116.26E,40.26N) | (114.18E,30.75N) | (121.91E,31.00N) | (113.72E,34.82N) | (120.83E,33.05N) |
|  | (114.47E,30.82N) | (113.87E,30.31N) | (121.88E,31.47N) | (113.73E,34.87N) | (119.39E,32.99N) |
|  | (130.58E,42.61N) | (115.61E,38.81N) | (115.95E,29.10N) | (114.50E,34.94N) | (119.33E,32.98N) |
|  | (117.15E,39.02N) | (115.38E,29.88N) | (115.95E,29.19N) | (116.63E,34.52N) | (119.34E,32.86N) |
|  | (115.92E,29.09N) | (117.05E,30.41N) | (116.69E,30.03N) | (119.75E,39.98N) | (118.48E,33.32N) |
|  | (118.88E,31.53N) | (117.09E,30.76N) | (114.63E,30.16N) | (119.52E,39.84N) | (118.52E,33.34N) |
|  | (116.35E,30.03N) | (117.89E,39.20N) | (117.46E,31.63N) | (122.28E,45.13N) | (111.15E,34.80N) |
|  | (118.94E,31.48N) | (120.44E,36.07N) | (119.62E,26.04N) | (122.25E,45.06N) | (111.13E,34.81N) |
|  | (116.49E,28.85N) | (116.69E,30.23N) | (118.79E,32.08N) | (126.60E,45.80N) | (132.84E,45.14N) |
|  | (116.41E,29.91N) | (119.06E,37.80N) | (113.87E,30.30N) | (123.61E,43.89N) | (123.57E,45.94N) |
|  | (120.41E,31.03N) | (115.98E,29.15N) | (114.24E,30.13N) | (123.29E,42.75N) | (121.74E,40.87N) |
|  | (117.35E,38.74N) | (118.21E,39.07N) | (122.46E,37.14N) | (123.24E,42.71N) | (121.79E,40.91N) |
|  | (120.08E,30.30N) | (115.94E,29.09N) | (114.73E,30.67N) | (130.61E,42.59N) | (124.36E,39.88N) |
|  | (115.85E,29.10N) | (116.46E,28.85N) | (113.55E,29.69N) | (130.54E,42.64N) | (124.26E,39.91N) |
|  | (120.34E,30.54N) | (117.99E,39.22N) | (117.16E,30.39N) | (123.38E,41.75N) | (108.89E,41.02N) |
|  | (116.47E,30.15N) | (119.56E,29.11N) | (119.62E,26.68N) | (122.85E,41.72N) | (108.85E,40.92N) |
|  | (117.42E,31.71N) | (120.42E,36.94N) | (120.07E,26.87N) | (121.56E,40.86N) | (86.88E,41.95N) |
|  | (116.20E,28.86N) | (115.67E,39.96N) | (117.36E,48.92N) | (121.27E,41.55N) | (87.28E,41.97N) |
|  | (119.47E,29.12N) | (114.20E,30.12N) | (117.62E,49.15N) | (104.33E,30.61N) | (124.22E,47.21N) |
|  | (115.83E,29.06N) | (120.84E,29.64N) | (115.94E,29.07N) | (104.08E,30.40N) | (124.21E,47.20N) |
|  | (116.29E,28.92N) | (119.54E,28.66N) | (115.94E,29.14N) | (103.86E,30.39N) | (124.23E,47.20N) |
|  | (121.17E,28.86N) | (114.85E,43.39N) | (116.05E,29.18N) | (105.45E,28.90N) | (124.23E,47.21N) |
|  | (121.93E,31.48N) | (120.81E,30.80N) | (100.11E,36.19N) | (104.79E,26.59N) | (132.33E,45.36N) |
|  | (120.58E,31.41N) | (116.22E,28.78N) | (116.57E,39.78N) | (109.19E,21.42N) | (132.42E,45.38N) |
|  | (116.59E,29.15N) | (120.19E,35.92N) | (116.48E,39.77N) | (110.00E,31.48N) | (132.39E,45.40N) |
|  | (119.39E,35.39N) | (116.25E,28.90N) | (116.47E,39.77N) | (112.80E,29.52N) | (123.53E,45.96N) |
|  | (118.96E,37.60N) | (119.57E,31.59N) | (116.32E,39.92N) | (112.83E,29.44N) | (123.53E,45.93N) |
|  | (119.38E,29.14N) | (120.44E,36.97N) | (116.22E,39.92N) | (112.87E,29.39N) | (123.67E,45.92N) |
|  | (117.10E,40.32N) | (118.78E,31.48N) | (116.22E,39.84N) | (112.88E,29.26N) | (123.61E,45.90N) |
|  | (117.78E,30.75N) | (104.22E,26.85N) | (116.20E,39.87N) | (113.05E,29.38N) | (124.17E,45.28N) |
|  | (120.51E,33.60N) | (118.87E,31.50N) | (116.27E,39.99N) | (112.45E,28.78N) | (124.33E,45.23N) |
|  | (117.33E,31.58N) | (119.02E,32.20N) | (116.18E,40.10N) | (113.46E,29.79N) | (122.03E,41.17N) |
|  | (117.66E,30.93N) | (100.10E,36.29N) | (116.31E,40.13N) | (112.28E,29.93N) | (122.05E,41.17N) |
|  | (117.30E,31.58N) | (109.89E,39.09N) | (116.32E,40.13N) | (114.69E,29.18N) | (121.99E,41.16N) |
|  | (116.50E,29.83N) | (114.25E,30.10N) | (116.71E,40.11N) | (118.28E,33.20N) | (104.09E,30.40N) |
|  | (120.74E,30.88N) | (116.43E,30.12N) | (116.67E,40.15N) | (118.37E,33.24N) | (116.94E,40.54N) |
|  | (117.19E,38.92N) | (118.36E,39.19N) | (117.00E,40.51N) | (118.37E,33.21N) | (118.11E,32.99N) |
|  | (116.46E,28.84N) |  |  |  |  |
| ***Cygnus olor*** | (81.59E,43.72N) | (81.23E,43.90N) | (87.35E,47.19N) | (120.44E,31.20N) | (120.90E,27.94N) |
|  | (86.64E,47.92N) | (81.41E,43.85N) | (99.21E,25.12N) | (117.13E,38.94N) | (115.36E,29.85N) |
|  | (81.88E,43.64N) | (81.37E,43.86N) | (118.69E,37.57N) | (108.72E,37.95N) | (111.49E,30.57N) |
|  | (82.91E,44.80N) | (80.92E,43.93N) | (117.42E,38.79N) | (102.33E,35.87N) | (117.46E,48.96N) |
|  | (83.09E,44.90N) | (81.33E,44.56N) | (118.96E,37.60N) | (108.75E,40.84N) | (117.06E,48.74N) |
|  | (82.92E,44.79N) | (81.31E,44.54N) | (116.31E,40.13N) | (117.58E,48.35N) | (117.51E,48.35N) |
|  | (82.87E,44.82N) | (82.64E,45.11N) | (118.96E,37.80N) | (108.88E,40.99N) | (117.44E,48.32N) |
|  | (83.24E,44.83N) | (82.41E,44.62N) | (123.60E,41.82N) | (108.79E,40.87N) | (117.37E,48.26N) |
|  | (82.83E,44.82N) | (82.43E,44.64N) | (119.10E,37.79N) | (80.60E,43.94N) | (117.78E,48.05N) |
|  | (82.94E,44.76N) | (82.42E,44.66N) | (113.72E,34.81N) | (121.12E,30.30N) | (126.59E,45.80N) |
|  | (82.88E,44.81N) | (82.43E,44.65N) | (120.42E,31.04N) | (121.39E,30.11N) | (122.33E,45.12N) |
|  | (82.87E,44.80N) | (82.31E,44.66N) | (116.57E,39.77N) | (117.07E,40.53N) | (123.32E,41.72N) |
|  | (82.82E,44.78N) | (80.88E,44.99N) | (116.27E,40.00N) | (116.62E,39.78N) | (122.89E,41.75N) |
|  | (82.92E,44.82N) | (81.10E,44.98N) | (115.77E,40.38N) | (116.46E,39.78N) | (122.88E,41.76N) |
|  | (82.70E,44.78N) | (81.03E,44.98N) | (120.58E,33.52N) | (116.03E,39.69N) | (119.71E,41.13N) |
|  | (82.78E,44.82N) | (81.04E,44.99N) | (117.52E,48.36N) | (116.01E,39.73N) | (114.98E,43.98N) |
|  | (83.26E,44.84N) | (80.90E,44.98N) | (116.26E,40.19N) | (116.22E,39.82N) | (115.10E,43.33N) |
|  | (83.01E,44.81N) | (81.41E,43.83N) | (120.80E,31.90N) | (116.22E,39.92N) | (113.57E,34.76N) |
|  | (82.81E,44.86N) | (80.57E,43.99N) | (120.45E,31.15N) | (115.85E,40.41N) | (111.15E,34.82N) |
|  | (82.77E,44.87N) | (80.67E,44.04N) | (85.95E,44.45N) | (115.81E,40.42N) | (110.02E,34.58N) |
|  | (82.62E,45.15N) | (80.66E,44.00N) | (116.97E,40.54N) | (117.39E,38.74N) | (112.52E,37.91N) |
|  | (82.79E,44.84N) | (80.86E,43.94N) | (117.53E,38.64N) | (117.56E,39.31N) | (108.94E,40.96N) |
|  | (81.62E,43.69N) | (80.77E,43.90N) | (115.83E,40.40N) | (118.36E,39.19N) | (100.68E,36.65N) |
|  | (81.61E,43.71N) | (80.79E,43.91N) | (117.11E,38.91N) | (119.32E,39.68N) | (99.94E,37.02N) |
|  | (81.50E,43.78N) | (80.62E,43.82N) | (114.88E,43.42N) | (119.08E,37.74N) | (102.27E,35.88N) |
|  | (81.42E,43.81N) | (81.19E,43.92N) | (117.50E,38.52N) | (118.75E,37.59N) | (100.10E,36.19N) |
|  | (81.45E,43.83N) | (80.67E,44.05N) | (86.84E,47.92N) | (118.82E,37.39N) | (87.03E,47.24N) |
|  | (81.55E,43.75N) | (80.59E,43.95N) | (100.72E,36.56N) | (118.77E,37.35N) | (87.04E,47.29N) |
|  | (81.57E,43.74N) | (81.32E,43.87N) | (87.44E,47.07N) | (119.04E,37.80N) | (87.45E,47.26N) |
|  | (81.57E,43.72N) | (81.45E,43.81N) | (87.25E,47.27N) | (119.03E,37.75N) | (87.44E,47.22N) |
|  | (82.19E,43.84N) | (81.64E,43.67N) | (112.54E,34.88N) | (118.71E,38.06N) | (87.40E,47.24N) |
|  | (82.47E,43.79N) | (81.00E,43.91N) | (116.26E,40.26N) | (121.64E,36.83N) | (87.53E,47.35N) |
|  | (82.08E,43.59N) | (87.50E,46.87N) | (88.44E,42.54N) | (120.11E,36.21N) | (87.49E,47.10N) |
|  | (82.50E,43.48N) | (87.09E,47.18N) | (114.04E,34.87N) | (119.44E,35.31N) | (87.06E,47.21N) |
|  | (82.13E,43.59N) | (80.68E,43.86N) | (111.15E,34.78N) | (120.40E,33.74N) | (87.37E,47.00N) |
|  | (82.01E,43.60N) | (81.27E,43.86N) | (118.66E,32.30N) | (120.52E,33.60N) | (87.37E,46.94N) |
|  | (82.19E,43.59N) | (81.96E,43.61N) | (117.68E,39.13N) | (117.07E,36.73N) | (88.38E,47.24N) |
|  | (82.21E,43.59N) | (81.91E,43.63N) | (99.90E,36.98N) | (116.81E,36.66N) | (82.61E,45.16N) |
|  | (81.83E,43.63N) | (87.29E,47.30N) | (118.98E,37.76N) | (118.76E,32.04N) | (100.47E,38.98N) |
|  | (81.47E,43.80N) | (118.97E,37.74N) | (117.47E,48.34N) | (120.45E,31.15N) | (81.02E,44.65N) |
|  | (81.64E,43.69N) | (106.35E,38.81N) | (117.12E,38.97N) | (120.44E,31.15N) | (81.07E,44.67N) |
|  | (80.64E,43.84N) | (108.87E,40.93N) | (93.85E,40.36N) | (120.06E,31.36N) | (81.05E,44.66N) |
|  | (80.56E,43.83N) | (119.08E,37.78N) | (119.02E,37.83N) | (120.81E,31.80N) | (80.89E,44.98N) |
|  | (80.66E,43.84N) | (81.27E,43.89N) | (117.42E,38.80N) | (121.16E,30.32N) | (83.62E,44.98N) |
|  | (81.13E,43.91N) | (117.35E,38.74N) | (121.57E,40.89N) | (121.37E,30.13N) | (81.64E,43.70N) |
|  | (81.09E,43.90N) | (116.55E,40.04N) | (121.49E,30.25N) | (121.05E,28.60N) | (81.64E,43.70N) |
|  | (82.72E,43.55N) | (81.52E,43.77N) | (81.43E,43.84N) | (80.48E,43.97N) |  |

**Table S2.** Occurrence records of three swan species in China, summarized by source and time period. Numbers are shown before filtering and after quality control (duplicate removal, coordinate verification, and spatial thinning).

| **Species** | **Data source** | **Time span** | **Records**  **(before filtering)** | **Records**  **(after filtering)** |
| --- | --- | --- | --- | --- |
| Whooper Swan | Satellite tracking | 2021$-$2022 | 385,865 | 52 |
|  | GBIF | 2004$-$2022 | 1657 | 370 |
|  | BirdReport | 2014$-$2024 | 265 | 206 |
|  | Field surveys | 2024 | 6 | 4 |
|  | Total | 2004$-$2024 | 387,793 | 632 |
| Bewick’s Swan | Satellite tracking | NA | NA | NA |
|  | GBIF | 2006$-$2023 | 811 | 552 |
|  | BirdReport | 2015$-$2022 | 35 | 29 |
|  | Field surveys | NA | NA | NA |
|  | Total | 2006$-$2023 | 846 | 581 |
| Mute Swan | Satellite tracking | 2021$-$2022 | 35,705 | 45 |
|  | GBIF | 2007$-$2022 | 179 | 158 |
|  | BirdReport | 2014$-$2023 | 31 | 23 |
|  | Field surveys | 2024 | 9 | 8 |
|  | Total | 2007$-$2024 | 35,924 | 234 |

**Table S3.** General Circulation Models (GCMs)

| **Global Climate Model** | **Description** |
| --- | --- |
| BCC-CSM2-MR | Beijing Climate Center-Climate System Model version 2-Medium Resolution |
| MIROC6 | Model for Interdisciplinary Research on Climate |
| HadGEM3-GC31-LL | Third Hadley Centre Global Environment Model in the Global Coupled Configuration 3.1 |

**Table S4.** All environmental variables used in this study

| **Classification** | **Variables** | **Description** | **Unit** |
| --- | --- | --- | --- |
| Bioclimate | WorldClim_Bio1 | Annual Mean Temperature | ℃ |
|  | WorldClim_Bio2 | Mean Diurnal Range  (Mean of monthly (max temp $-$ min temp)) | ℃ |
|  | WorldClim_Bio3 | Isothermality  (BIO2/BIO7) ($\times$100) | - |
|  | WorldClim_Bio4 | Temperature Seasonality  (standard deviation $\times$100) | - |
|  | WorldClim_Bio5 | Max Temperature of Warmest Month | ℃ |
|  | WorldClim_Bio6 | Min Temperature of Coldest Month | ℃ |
|  | WorldClim_Bio7 | Temperature Annual Range (BIO5$-$BIO6) | ℃ |
|  | WorldClim_Bio8 | Mean Temperature of Wettest Quarter | ℃ |
|  | WorldClim_Bio9 | Mean Temperature of Driest Quarter | ℃ |
|  | WorldClim_Bio10 | Mean Temperature of Warmest Quarter | ℃ |
|  | WorldClim_Bio11 | Mean Temperature of Coldest Quarter | ℃ |
|  | WorldClim_Bio12 | Annual Precipitation | mm |
|  | WorldClim_Bio13 | Precipitation of Wettest Month | mm |
|  | WorldClim_Bio14 | Precipitation of Driest Month | mm |
|  | WorldClim_Bio15 | Precipitation Seasonality  (Coefficient of Variation) | - |
|  | WorldClim_Bio16 | Precipitation of Wettest Quarter | mm |
|  | WorldClim_Bio17 | Precipitation of Driest Quarter | mm |
|  | WorldClim_Bio18 | Precipitation of Warmest Quarter | mm |
|  | WorldClim_Bio19 | Precipitation of Coldest Quarter | mm |
| Topographic factor | DEM | Relative Vertical Height | m |
|  | Slope | Surface Slope Degree | °/% |
|  | Aspect | Terrain Aspect | ° |
| Vegetation factor | NDVI | Normalized Difference Vegetation Index | - |
| Human activity | HFP | Human Footpoint | - |
| Water source | Dis-water | The distance from water source | km |

**Table S5.** Selected environmental variables used in the MaxEnt models for three swan species

| **Species** | **Category** | **Variables** | **Description** | **Unit** |
| --- | --- | --- | --- | --- |
| Whooper Swan | Bioclimate | Bio2 | Mean Diurnal Range (Mean of monthly (max temp $-$ min temp)) | ℃ |
|  |  | Bio3 | Isothermality (BIO2/BIO7) ($\times$100) | - |
|  |  | Bio4 | Temperature Seasonality  (standard deviation $\times$100) | - |
|  |  | Bio5 | Max Temperature of Warmest Month | ℃ |
|  |  | Bio9 | Mean Temperature of Driest Quarter | ℃ |
|  |  | Bio13 | Precipitation of Wettest Month | mm |
|  |  | Bio14 | Precipitation of Driest Month | mm |
|  |  | Bio15 | Precipitation Seasonality (Coefficient of Variation) | mm |
|  | Topographic factor | DEM | Relative Vertical Height | m |
|  |  | Slope | Surface Slope Degree | °/% |
|  |  | Aspect | Terrain Aspect | ° |
|  | Vegetation factor | NDVI | Normalized Difference Vegetation Index | - |
|  | Human activity | HFP | Human Footprint | - |
|  | Water source | Dis-water | Distance to Water Sources | km |
| Bewick's Swan | Bioclimate | Bio3 | Isothermality (BIO2/BIO7) ($\times$100) | - |
|  |  | Bio5 | Max Temperature of Warmest Month | ℃ |
|  |  | Bio7 | Temperature Annual Range (BIO5$-$BIO6) | ℃ |
|  |  | Bio17 | Precipitation of Driest Quarter | mm |
|  | Topographic factor | DEM | Relative Vertical Height | m |
|  |  | Slope | Surface Slope Degree | °/% |
|  |  | Aspect | Terrain Aspect | ° |
|  | Vegetation factor | NDVI | Normalized Difference Vegetation Index | - |
|  | Human activity | HFP | Human Footprint | - |
|  | Water source | Dis-water | Distance to Water Sources | km |
| Mute Swan | Bioclimate | Bio2 | Mean Diurnal Range (Mean of monthly (max temp $-$ min temp)) | ℃ |
|  |  | Bio3 | Isothermality (BIO2/BIO7) ($\times$100) | - |
|  |  | Bio5 | Max Temperature of Warmest Month | ℃ |
|  |  | Bio8 | Mean Temperature of Wettest Quarter | ℃ |
|  |  | Bio9 | Mean Temperature of Driest Quarter | ℃ |
|  |  | Bio12 | Annual Precipitation | mm |
|  |  | Bio19 | Precipitation of Coldest Quarter | mm |
|  | Topographic factor | Slope | Surface Slope Degree | °/% |
|  |  | Aspect | Terrain Aspect | ° |
|  | Vegetation factor | NDVI | Normalized Difference Vegetation Index | - |
|  | Human activity | HFP | Human Footprint | - |
|  | Water source | Dis-water | Distance to Water Sources | km |

**Table S6.** Uncertainty analysis of predicted suitable habitat areas (mean area $\pm$ SD, min, max, and CV%) for three swan species (Whooper Swan, Bewick’s Swan, and Mute Swan) under different SSP scenarios in the 2030s and 2050s.

| **Species** | **Period** | **Scenario** | **Suitability** | **Mean area**  **(×10^4^ km²)** | **SD** | **Min**  **(×10^4^ km²)** | **Max**  **(×10^4^ km²)** | **CV**  **(%)** |
| --- | --- | --- | --- | --- | --- | --- | --- | --- |
| Whooper Swan | 2030s | SSP1$-$2.6 | High | 4.12 | 0.33 | 3.81 | 4.46 | 7.91 |
|  |  |  | Moderate | 13.76 | 0.82 | 13.08 | 14.67 | 5.97 |
|  |  |  | Low | 36.36 | 2.82 | 33.56 | 39.19 | 7.74 |
|  |  | SSP2$-$4.5 | High | 4.25 | 0.20 | 4.02 | 4.41 | 4.77 |
|  |  |  | Moderate | 12.82 | 1.05 | 11.82 | 13.91 | 8.17 |
|  |  |  | Low | 40.93 | 2.60 | 38.29 | 43.49 | 6.35 |
|  |  | SSP5$-$8.5 | High | 3.98 | 0.35 | 3.58 | 4.2 | 8.78 |
|  |  |  | Moderate | 12.35 | 0.18 | 12.15 | 12.48 | 1.44 |
|  |  |  | Low | 39.90 | 3.11 | 36.51 | 42.61 | 7.78 |
|  | 2050s | SSP1$-$2.6 | High | 3.91 | 0.30 | 3.61 | 4.21 | 7.67 |
|  |  |  | Moderate | 12.29 | 0.67 | 11.71 | 13.02 | 5.44 |
|  |  |  | Low | 41.07 | 4.26 | 36.48 | 44.9 | 10.37 |
|  |  | SSP2$-$4.5 | High | 3.97 | 0.28 | 3.69 | 4.24 | 6.93 |
|  |  |  | Moderate | 13.06 | 0.98 | 11.96 | 13.86 | 7.54 |
|  |  |  | Low | 41.80 | 1.96 | 39.55 | 43.16 | 4.70 |
|  |  | SSP5$-$8.5 | High | 4.01 | 0.12 | 3.88 | 4.1 | 2.92 |
|  |  |  | Moderate | 13.20 | 0.71 | 12.72 | 14.01 | 5.34 |
|  |  |  | Low | 35.64 | 3.32 | 31.88 | 38.16 | 9.31 |
| Bewick's Swan | 2030s | SSP1$-$2.6 | High | 5.51 | 0.06 | 5.46 | 5.57 | 1.03 |
|  |  |  | Moderate | 11.12 | 0.44 | 10.63 | 11.49 | 3.99 |
|  |  |  | Low | 47.12 | 4.36 | 43.34 | 51.89 | 9.25 |
|  |  | SSP2$-$4.5 | High | 5.29 | 0.18 | 5.15 | 5.49 | 3.40 |
|  |  |  | Moderate | 11.16 | 0.23 | 10.98 | 11.42 | 2.08 |
|  |  |  | Low | 54.02 | 3.52 | 50.88 | 57.83 | 6.52 |
|  |  | SSP5$-$8.5 | High | 5.44 | 0.10 | 5.33 | 5.51 | 1.77 |
|  |  |  | Moderate | 10.87 | 0.81 | 10.05 | 11.67 | 7.45 |
|  |  |  | Low | 45.58 | 1.52 | 44.16 | 47.18 | 3.33 |
|  | 2050s | SSP1$-$2.6 | High | 5.81 | 0.26 | 5.52 | 6.01 | 4.45 |
|  |  |  | Moderate | 10.29 | 0.64 | 9.77 | 11.01 | 6.24 |
|  |  |  | Low | 47.45 | 0.71 | 46.80 | 48.20 | 1.49 |
|  |  | SSP2$-$4.5 | High | 5.52 | 0.46 | 5.11 | 6.02 | 8.34 |
|  |  |  | Moderate | 10.36 | 0.31 | 10.01 | 10.59 | 2.96 |
|  |  |  | Low | 48.43 | 4.74 | 45.41 | 53.89 | 9.79 |
|  |  | SSP5$-$8.5 | High | 5.72 | 0.25 | 5.49 | 5.98 | 4.32 |
|  |  |  | Moderate | 10.70 | 0.50 | 10.13 | 11.02 | 4.63 |
|  |  |  | Low | 50.27 | 4.50 | 46.07 | 55.02 | 8.95 |
| Mute Swan | 2030s | SSP1$-$2.6 | High | 3.71 | 0.16 | 3.55 | 3.86 | 4.19 |
|  |  |  | Moderate | 9.87 | 0.35 | 9.58 | 10.25 | 3.50 |
|  |  |  | Low | 46.23 | 1.58 | 45.05 | 48.02 | 3.41 |
|  |  | SSP2$-$4.5 | High | 3.55 | 0.31 | 3.31 | 3.90 | 8.68 |
|  |  |  | Moderate | 9.49 | 1.00 | 8.68 | 10.61 | 10.58 |
|  |  |  | Low | 45.24 | 6.48 | 38.31 | 51.15 | 14.33 |
|  |  | SSP5$-$8.5 | High | 3.33 | 0.19 | 3.12 | 3.45 | 5.55 |
|  |  |  | Moderate | 9.20 | 0.55 | 8.61 | 9.70 | 5.99 |
|  |  |  | Low | 57.43 | 6.71 | 52.86 | 65.13 | 11.68 |
|  | 2050s | SSP1$-$2.6 | High | 3.77 | 0.17 | 3.59 | 3.92 | 4.41 |
|  |  |  | Moderate | 10.37 | 0.67 | 9.65 | 10.96 | 6.42 |
|  |  |  | Low | 47.93 | 3.40 | 44.15 | 50.76 | 7.10 |
|  |  | SSP2$-$4.5 | High | 3.71 | 0.18 | 3.51 | 3.86 | 4.83 |
|  |  |  | Moderate | 9.99 | 0.68 | 9.25 | 10.59 | 6.81 |
|  |  |  | Low | 46.51 | 6.14 | 39.51 | 51.01 | 13.21 |
|  |  | SSP5$-$8.5 | High | 3.86 | 0.13 | 3.72 | 3.95 | 3.24 |
|  |  |  | Moderate | 10.03 | 0.37 | 9.66 | 10.40 | 3.69 |
|  |  |  | Low | 55.84 | 3.86 | 51.82 | 59.52 | 6.91 |

**Table S7.** Total conservation rates (%) under three thresholds (MTSS, 10% TP, ESS) for each species, scenario and period, and their range (max-min).

| Species | Shared Socioeconomic Pathways | Period | MTSS | 10% TP | ESS | Range  (max-min) |
| --- | --- | --- | --- | --- | --- | --- |
| Whooper Swan | - | recent | 9.43 | 10.58 | 10.00 | 1.15 |
|  | SSP1$-$2.6 | 2030s | 8.80 | 9.60 | 9.22 | 0.79 |
|  |  | 2050s | 8.85 | 10.05 | 9.39 | 1.20 |
|  | SSP2$-$4.5 | 2030s | 8.82 | 10.21 | 9.64 | 1.39 |
|  |  | 2050s | 9.43 | 10.32 | 10.02 | 0.89 |
|  | SSP5$-$8.5 | 2030s | 9.19 | 10.47 | 9.98 | 1.29 |
|  |  | 2050s | 8.82 | 9.82 | 9.22 | 1.00 |
| Bewick’s Swan | - | recent | 6.46 | 7.72 | 7.28 | 1.27 |
|  | SSP1$-$2.6 | 2030s | 5.52 | 6.66 | 6.14 | 1.15 |
|  |  | 2050s | 5.65 | 6.96 | 6.46 | 1.31 |
|  | SSP2$-$4.5 | 2030s | 5.36 | 6.67 | 6.17 | 1.32 |
|  |  | 2050s | 5.73 | 7.05 | 6.57 | 1.31 |
|  | SSP5$-$8.5 | 2030s | 5.78 | 6.85 | 6.43 | 1.07 |
|  |  | 2050s | 5.44 | 6.90 | 6.31 | 1.46 |
| Mute Swan | - | recent | 9.22 | 10.37 | 9.49 | 1.15 |
|  | SSP1$-$2.6 | 2030s | 9.91 | 10.23 | 9.84 | 0.38 |
|  |  | 2050s | 9.80 | 9.88 | 9.58 | 0.30 |
|  | SSP2$-$4.5 | 2030s | 9.63 | 9.96 | 9.48 | 0.48 |
|  |  | 2050s | 9.32 | 9.55 | 9.19 | 0.36 |
|  | SSP5$-$8.5 | 2030s | 9.75 | 10.18 | 9.86 | 0.44 |
|  |  | 2050s | 10.32 | 10.59 | 10.37 | 0.28 |


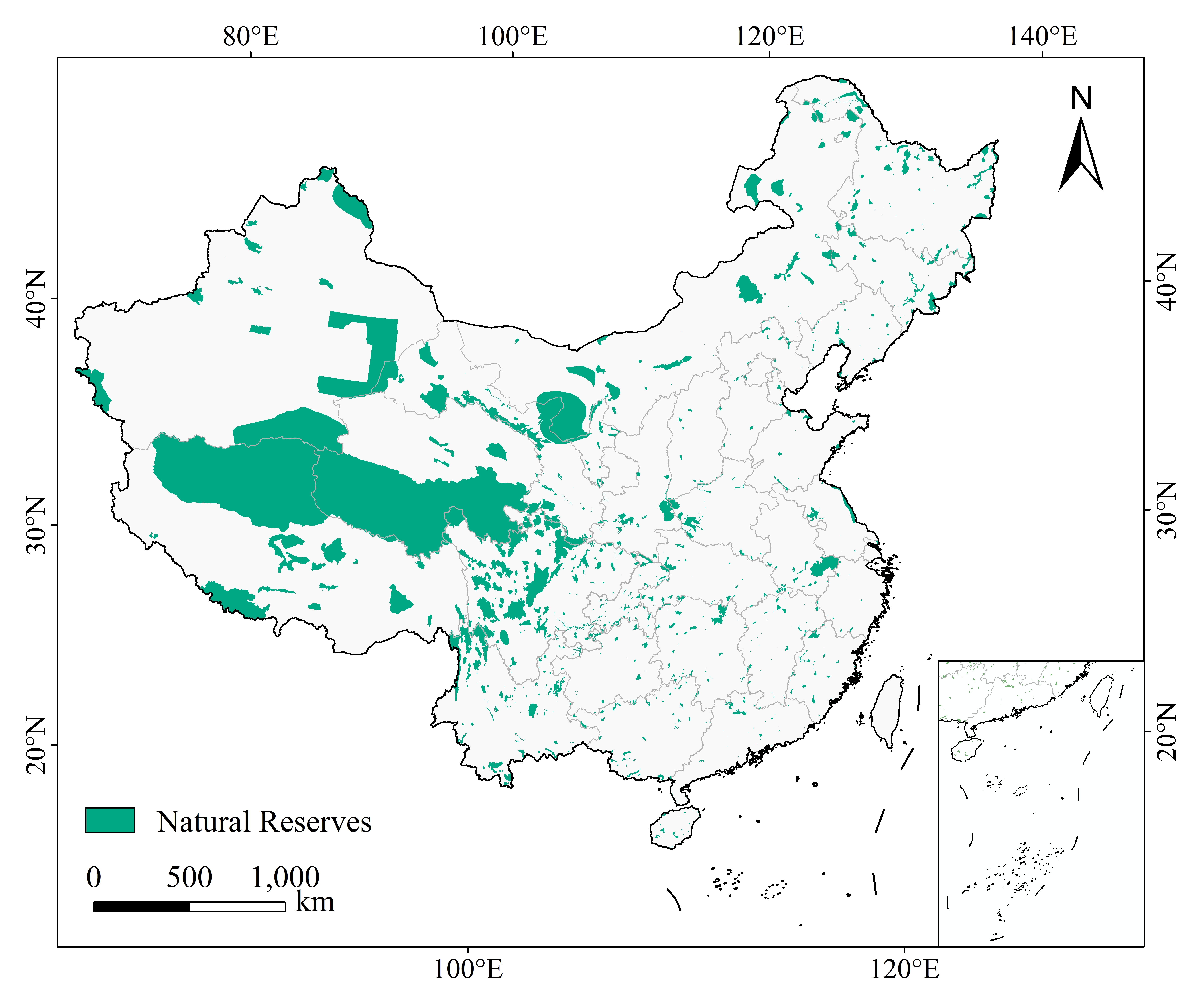


**Figure S1.** Distribution of protected areas in China

**
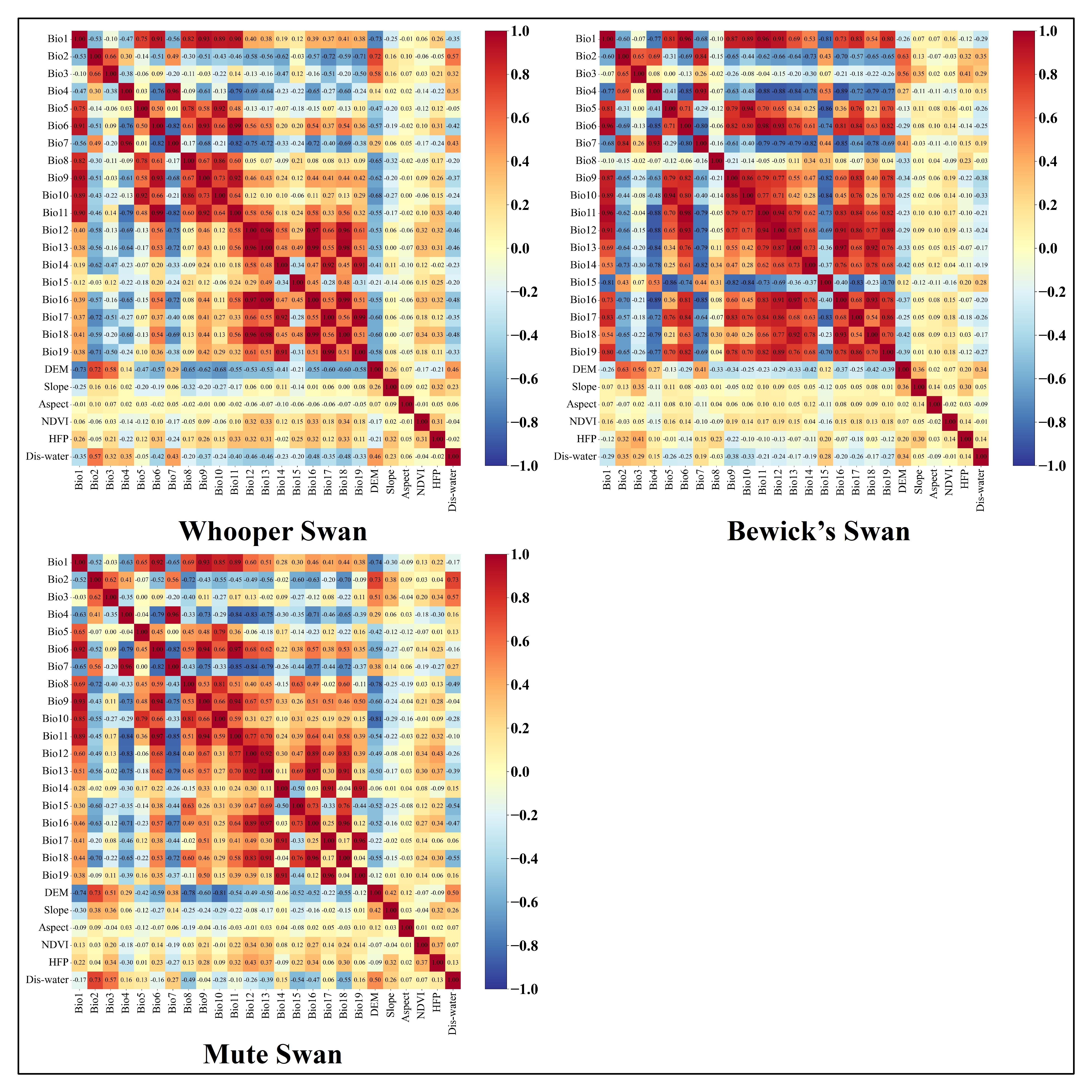
**

**Figure S2.** Spearman correlation heat maps of environmental variables used in constructing the MaxEnt models for Whooper Swan, Bewick’s Swan, and Mute Swan.

**Figure S3.** Distribution of suitable habitats for the three swan species in China under different future climate scenarios


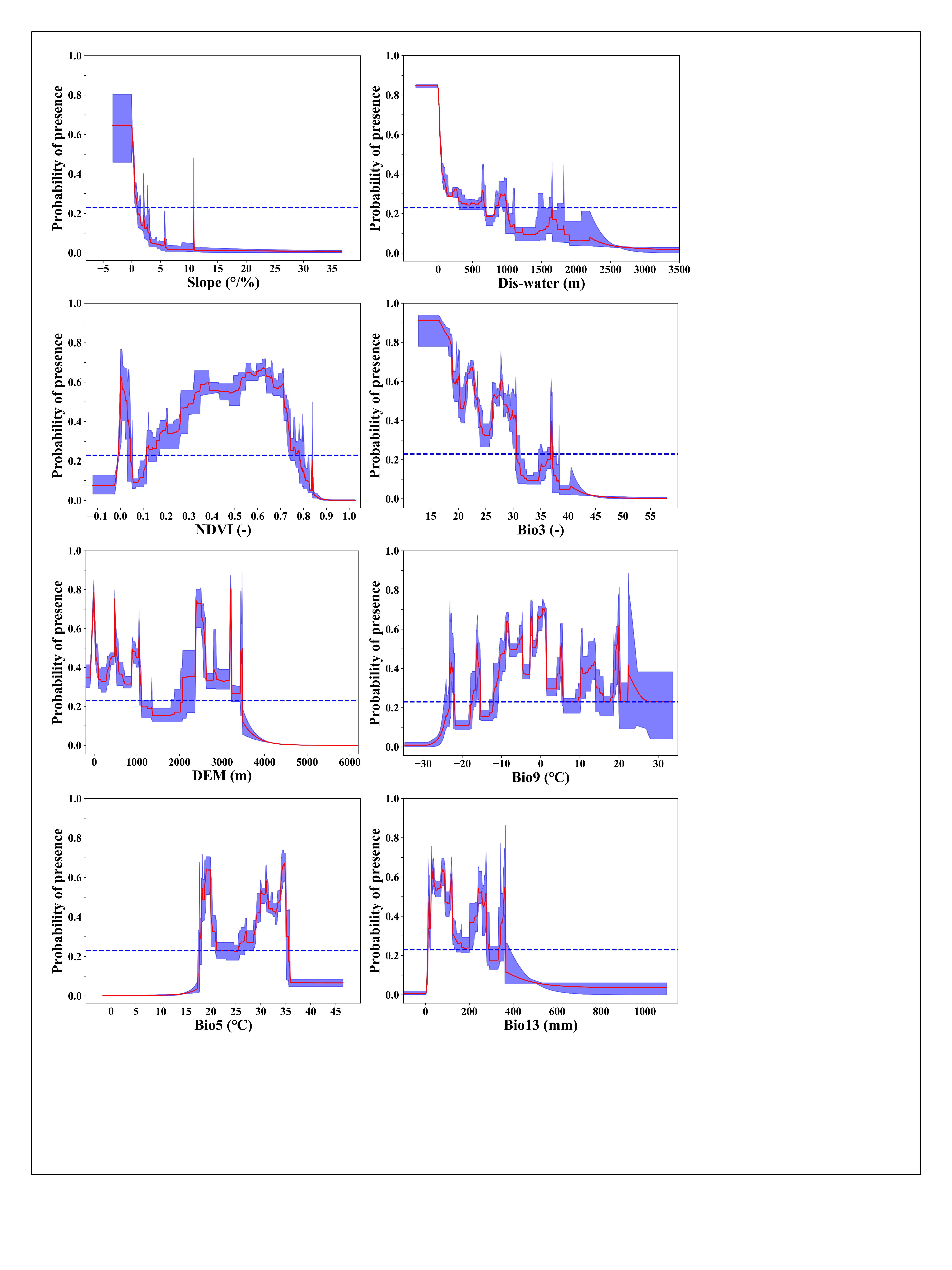


**Figure S4.** Response curves of the key environmental variables influencing the suitable habitat distribution of the Whooper Swan, including Slope, Dis-water, NDVI, Bio3, DEM, Bio9, Bio5, and Bio13, with a cumulative contribution exceeding 85%. The x-axis represents variable values, and the y-axis indicates the predicted probability of species presence. The red curve shows the mean response across 10 replicate runs, while the blue shading represents $\pm$1 standard deviation. The blue dashed line denotes the maximum training sensitivity plus specificity threshold (MTSS $=$ 0.2297), above which areas are considered suitable for species presence.


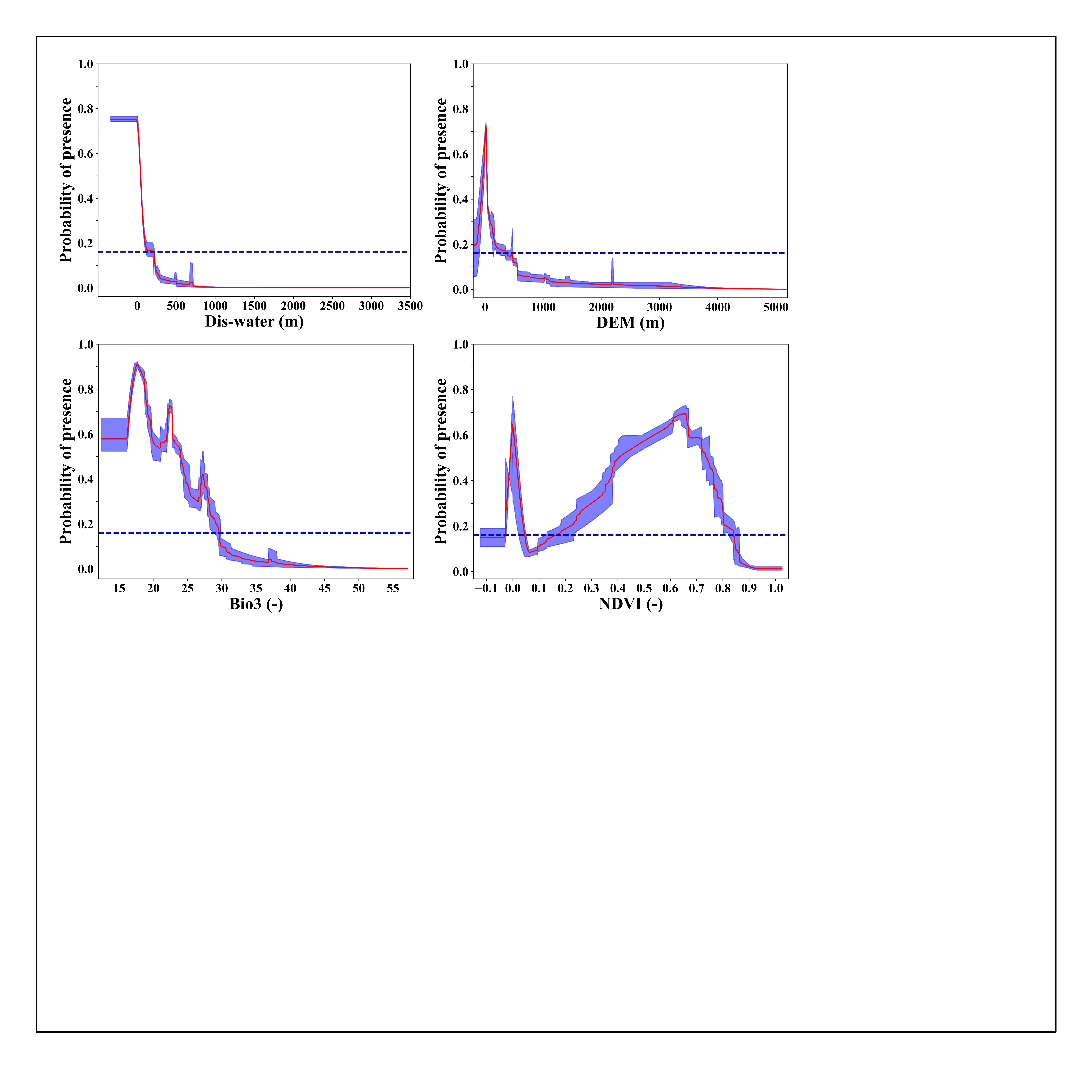


**Figure S5.** Response curves of the key environmental variables influencing the suitable habitat distribution of the Bewick's Swan, including Dis-water, DEM, Bio3 and NDVI, with a cumulative contribution exceeding 85%. The x-axis represents variable values, and the y-axis indicates the predicted probability of species presence. The red curve shows the mean response across 10 replicate runs, while the blue shading represents $\pm$1 standard deviation. The blue dashed line denotes the maximum training sensitivity plus specificity threshold (MTSS = 0.1611), above which areas are considered suitable for species presence.


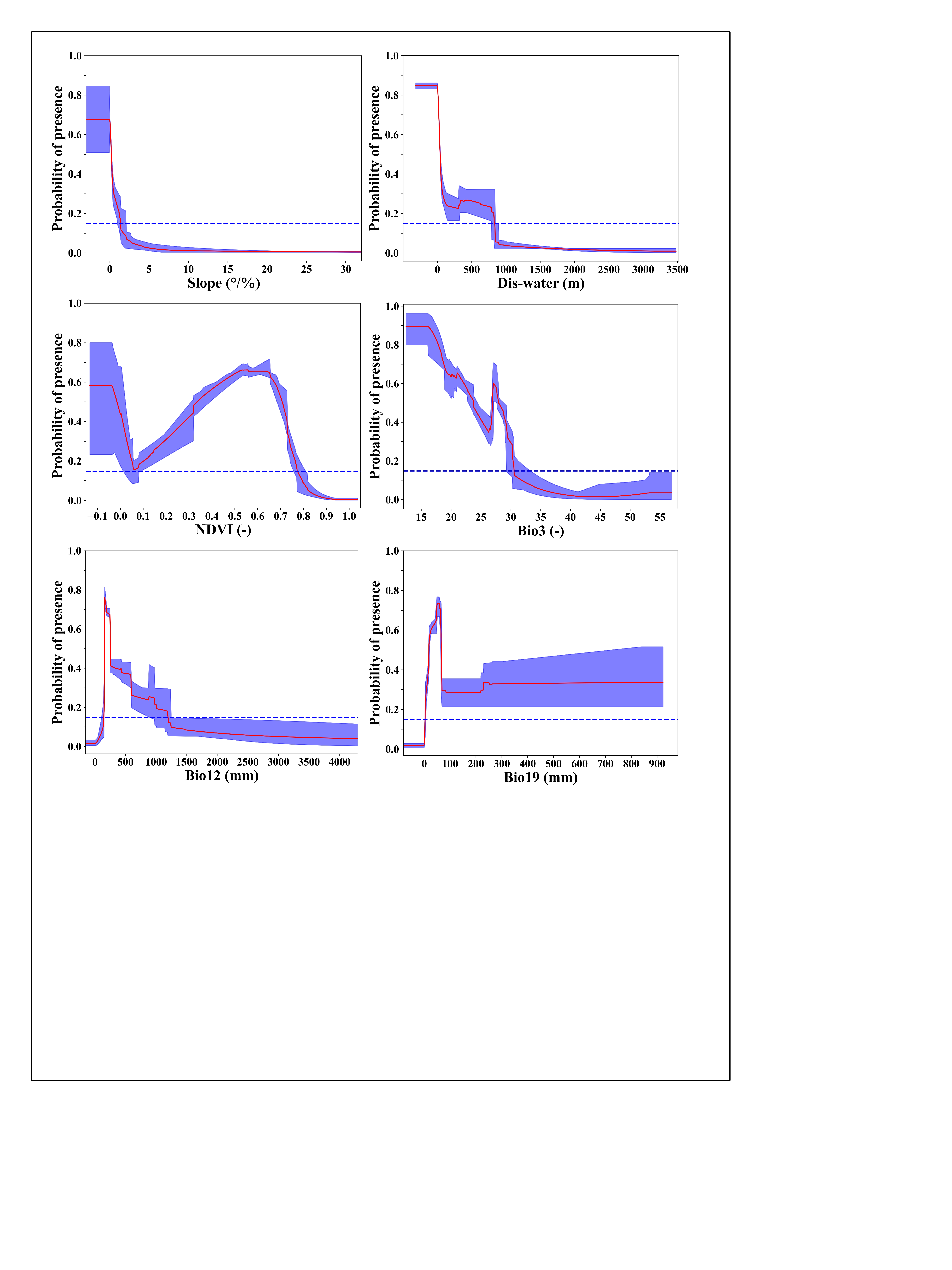


**Figure S6.** Response curves of the key environmental variables influencing the suitable habitat distribution of the Mute Swan, including Slope, Dis-water, NDVI, Bio3, Bio12 and Bio19, with a cumulative contribution exceeding 85%. The x-axis represents variable values, and the y-axis indicates the predicted probability of species presence. The red curve shows the mean response across 10 replicate runs, while the blue shading represents $\pm$1 standard deviation. The blue dashed line denotes the maximum training sensitivity plus specificity threshold (MTSS $=$ 0.1485), above which areas are considered suitable for species presence.
